# Reassessment of the enigmatic ruminant Miocene genus *Amphimoschus* Bourgeois, 1873 (Mammalia, Artiodactyla, Pecora)

**DOI:** 10.1371/journal.pone.0244661

**Published:** 2021-01-29

**Authors:** Bastien Mennecart, Grégoire Métais, Loïc Costeur, Léonard Ginsburg, Gertrud E. Rössner

**Affiliations:** 1 Naturhistorisches Museum Basel, Basel, Switzerland; 2 Naturhistorisches Museum Wien, Vienna, Austria; 3 CR2P - Centre de Recherche en Paléontologie – Paris, UMR 7207, MNHN – CNRS - Sorbonne Universités, Muséum National d’Histoire Naturelle, CP38, Paris, France; 4 Staatliche Naturwissenschaftliche Sammlungen Bayerns - Bayerische Staatssammlung für Paläontologie und Geologie, Munich, Germany; 5 Department für Geo- und Umweltwissenschaften, Ludwig-Maximilians-Universität München, Munich, Germany; Royal Belgian Institute of Natural Sciences, BELGIUM

## Abstract

*Amphimoschus* is an extinct Eurasian ruminant genus, mostly recorded in Europe, without a close living relative and, hence, an unknown systematic position. This genus is known from around 50 localities from the late early to the middle Miocene. Two species were described during 180 years, but since their first description during the late 19^th^ century and early 20^th^ century, hardly any detailed taxonomic work has been done on the genus. Over the years, extensive collecting and excavating activities have enriched collections with more and more complete material of this still rare and enigmatic animal. Most interestingly, a number of skull remains have been unearthed and are promising in terms of providing phylogenetic information. In the present paper, we describe cranial material, the bony labyrinth, the dentition through 780 teeth and five skulls from different ontogenetic stages. We cannot find a clear morphometric distinction between the supposedly smaller and older species *Amphimoschus artenensis* and the supposedly younger and larger species *A*. *ponteleviensis*. Accordingly, we have no reason to retain the two species and propose, following the principle of priority (ICZN chapter 6 article 23), that only *A*. *ponteleviensis* Bourgeois, 1873 is valid. Our studies on the ontogenetic variation of *Amphimoschus* does reveal that the sagittal crest may increase in size and a supraorbital ridge may appear with age. Despite the abundant material, the family affiliation is still uncertain.

## Introduction

The late early Miocene was a time period of huge changes in the European mammalian communities. A deep faunal renewal associated with a peak of first appearance data at the genus and family levels is observed during the MN3 biozone (early Orleanian, from ca. 19.5 to 17.2 Mya; e.g., [1,2]). A tectonically driven land bridge emerged between Eurasia, Africa and Anatolia permitting large-scale dispersals to occur [1–3]. Western European ruminants were strongly impacted by the event, with a renewal of about 80% of their generic diversity [1,2]. From MN3 onwards, modern ruminant families replaced their ancestral forerunners including pecorans without cranial appendages: *Amphitragulus*, *Dremotherium*, and *Bedenomeryx* [2]. While most Orleanian taxa can be attributed to well-known families (e.g., for MN3 Tragulidae, Cervidae, Palaeomerycidae, followed by Bovidae and Moschidae in MN4), *Amphimoschus* Bourgeois, 1873 [4], a stem pecoran ruminant that first appeared in Western Europe at that time, remains enigmatic. Most ruminant newcomers are known from their diagnostic cranial appendages, i.e., a new feature of ruminants appearing at that time, but *Amphimoschus* Bourgeois, 1873 [4] remains with a primitive aspect without horns or antlers. Yet, *Amphimoschus* Bourgeois, 1873 [4] does show higher crowned teeth, a derived condition, in comparison to most other modern ruminants known from the early to middle Miocene. The origin of crown groups in pecoran ruminants is still highly debated and the cranial appendages are generally used to differentiate the various families (e.g., [5]). The presence of many derived features of cranial and dental morphology, in combination with the lack of headgear, make this ruminant a peculiar taxon, which provides insights into the explosion of ruminant diversity at the early to middle Miocene transition.

**Fig 1:**
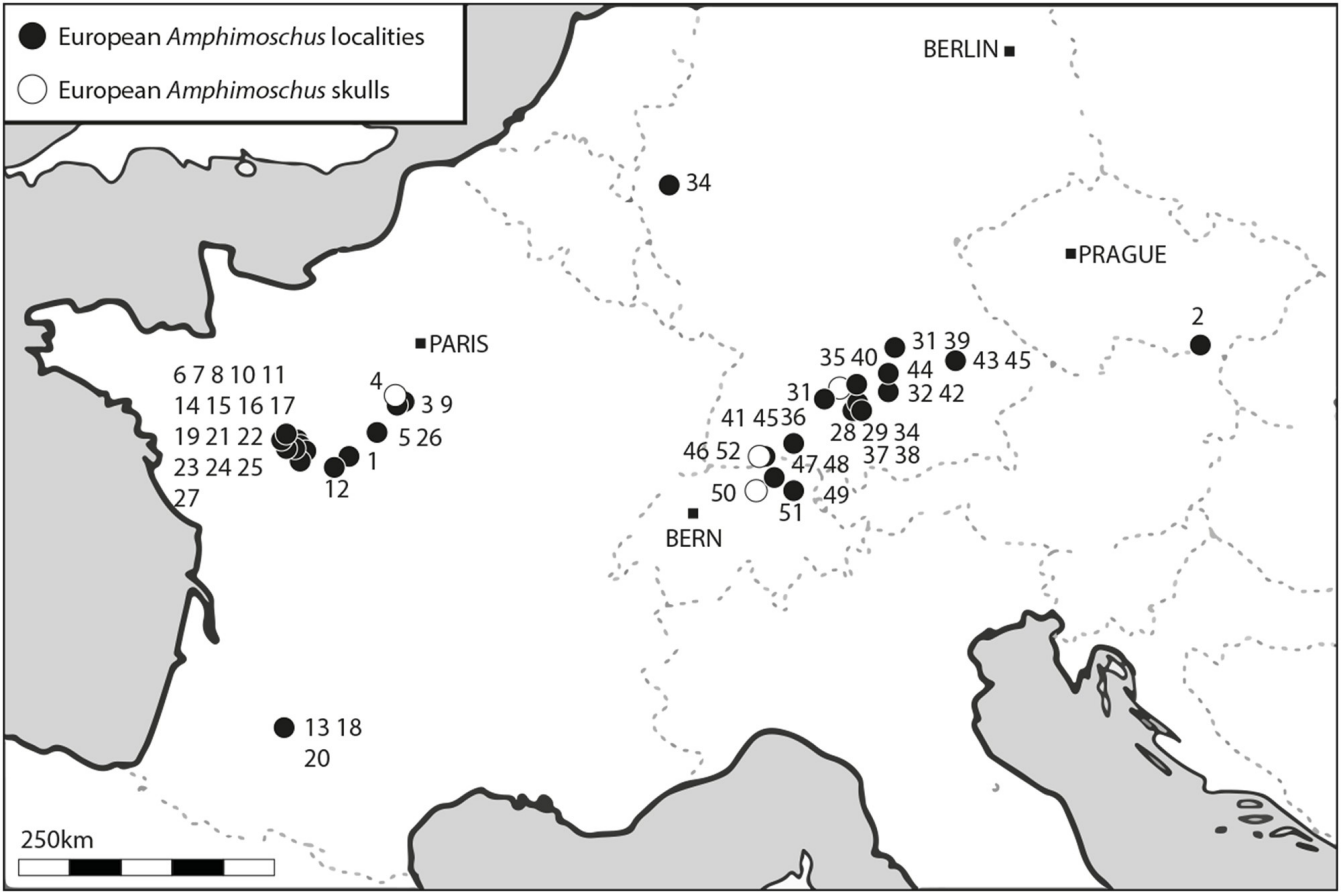
Location of the European *Amphimoschus* fossils. Corresponding localities are summarised in Table 1.

**Fig 2:**
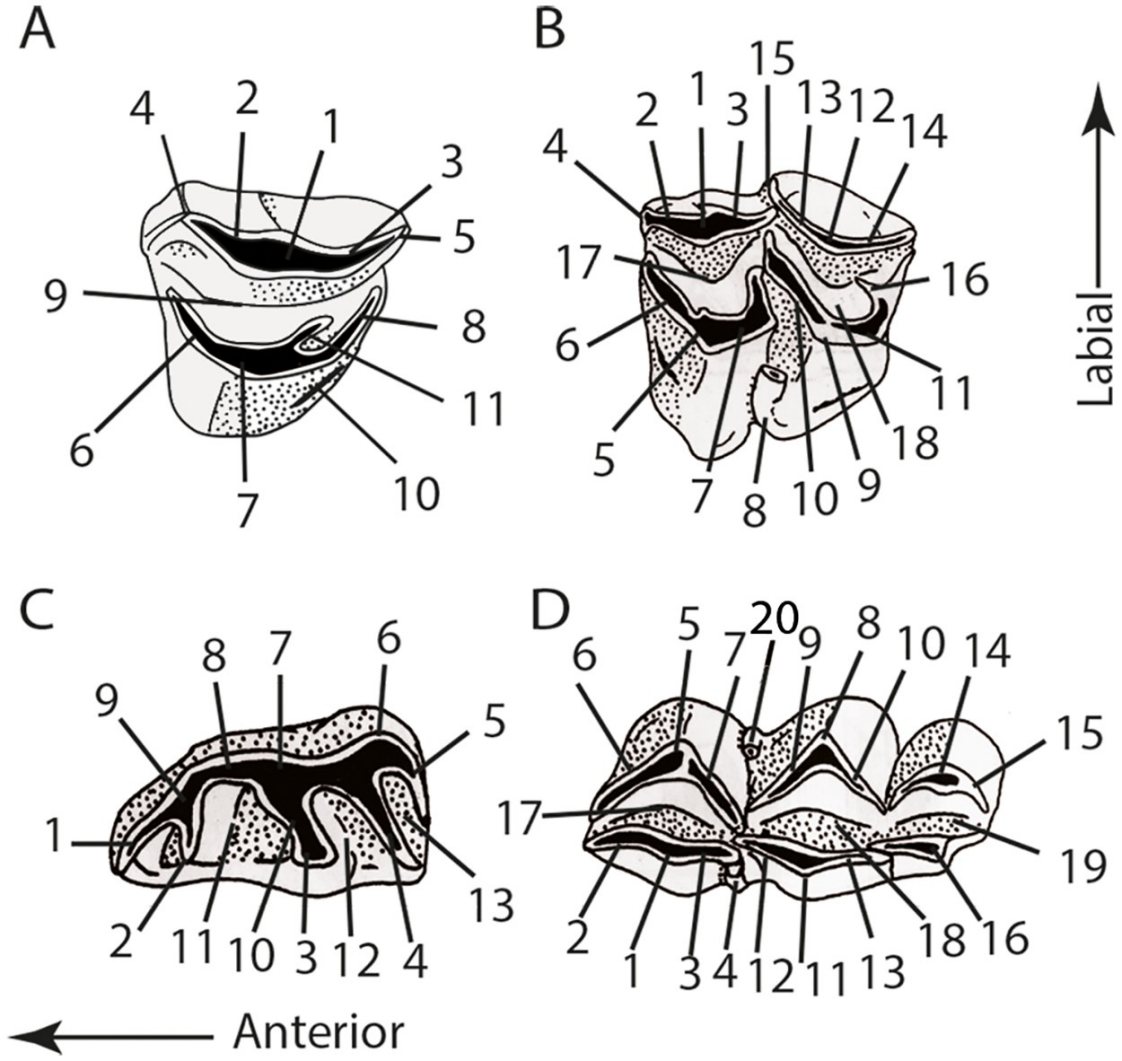
Dental nomenclature following Bärmann and Rössner [38]. Drawings of the molars and the lower premolar come from Gentry et al. [32] (Fig 23.15). **A**, Upper premolars. 1 anterolabial cone; 2 anterolabial crista; 3 posterolabial crista; 4 anterior style; 5 posterior style; 6 anterolingual crista; 7 lingual cone; 8 posterolingual crista; 9 fossa; 10 posterolingual cingulum; 11 central fold. **B**, Upper molars. 1 paracone; 2 preparacrista; 3 postparacrista; 4 parastyle; 5 protocone; 6 preprotocrista; 7 postprotocrista; 8 entostyle; 9 metaconule; 10 premetaconulecrista; 11 postmetaconulecrista; 12 metacone; 13 premetacrista; 14 postmetacrista; 15 mesostyle; 16 metaconule fold; 17 anterior fossa; 18 posterior fossa. **C**, Lower premolars. 1 anterior stylid; 2 anterior conid; 3 mesolingual conid; 4 posterolingual conid; 5 posterior stylid; 6 posterolabial conid; 7 posterolabial cristid; 8 mesolabial conid; 9 anterolabial cristid; 10 transverse cristid; 11 anterior valley; 12 posterior valley; 13 back valley. **D**, Lower molars. 1 metaconid; 2 premetacristid; 3 postmetacristid; 4 metastylid; 5 protoconid; 6 preprotocristid; 7 internal postprotocristid; 8 hypoconid; 9 prehypocristid; 10 posthypocristid; 11 entoconid; 12 preentocristid; 13 postentocristid; 14 hypoconulid; 15 posthypoconulidcristid; 16 entoconulid; 17 anterior fossa; 18 posterior fossa; 19 back fossa of m3; 20 ectostylid.

*Amphimoschus* Bourgeois, 1873 [4] is middle sized weighing ca. 40 kg [6], similar in size to the extant gerenuk or springbok [7]. It is currently defined by two different species: *Amphimoschus ponteleviensis* Bourgeois, 1873 [4] from the Sables et Marnes du Blésois Formation in Thenay (France) [8], but often reported as found in the Falun Formation in Pontlevoy (e.g. [9] and younger articles) and *Amphimoschus artenensis* Mayet, 1908 [9] from the Sables de l’Orléanais Formation in Artenay (France). This genus is well-known from Western and Central European localities dated from the late early to early middle Miocene, an interval encompassing the Mid-Miocene Climatic Optimum, in Germany (e.g. Eggingen-Mittelhart, Erkertshofen 2, Hambach 6, Langenau 1, Petersbuch 2), France (e.g. Artenay, Chilleurs aux bois, Esvres, La Brosse, Pellécahus, Pont-Boutard, Pontlevoy), and Switzerland (e.g. Benken, Käpfnach, Wildensbuch) (see Fig 1; [2,10,11], Table 1 for a complete list of localities). These localities document diverse depositional environments (swamps, lakes, rivers, karstic infillings, and marine deposits). More recently, the genus has been reported in Jiangsu [12] and Xishuigou [13,14] in China. Nevertheless, *Amphimoschus* spp. is often only mentioned in faunal lists because no detailed definition of these two species has been provided so far. *Amphimoschus artenensis* appears to be a little smaller and older than the type species *A*. *ponteleviensis* [10,15–17], even if some overlap in size and stratigraphic range exists [18]. A third species, *Cervus lunatus* described by von Meyer [19] and based on the material from Käpfnach (MN5), is considered by Stehlin [18] as a senior synonym of *A*. *ponteleviensis*, or at least congeneric. Moreover, Stehlin [18] could not detect significant size differences between the material of Käpfnach and that from older localities.

**Table 1:** European *Amphimoschus* localities.

	Type locality				Czech Republic		
1	*Thenay	MN5a	[4]	2	Spolocenstvo	e. Mio.	[11,100]
s	France						
3	Aérotrain	MN4b	This study	16	Meigné-le-Vicomte	MN5	[41]
4	Artenay (Chevilly)	MN4a	[10,32]	17	Méon	MN5	[41]
5	Beaugency	MN5	[101], This study	18	Montréal-du-Gers	MN4	This study
6	Breil	MN5	[41]	19	Noyant-sous-le-L.	MN5	[41]
7	Channay-sur-L.	MN5	[41]	20	Pellecahus	MN4a	[71]
8	Chavaignes	MN5	[41]	21	Pont-Boutard	MN5	[41]
9	Chilleur-aux-Bois	MN3	[18,102]	22	Pontigné	MN5	[41]
10	Cléré-les-Pins	MN5	[41]	23	Rillé	MN5	[41]
11	Genneteil	MN5	[41]	24	Saint-Laurent-le-Lin	MN5	[41]
12	Homme	MN5	[41]	25	Savigné-sur-Lathan	MN5	[41]
13	La Romieu	MN4	[15,102]	26	Tavers	MN5	This study
14	Lasse	MN5	[41]	27	Tourelet	MN5	[78]
15	Lublé	MN5	[41]				
	Germany						
28	Burg-Balzhausen	MN5	[75]	37	Mutterhofen	-	This study
29	Dechbetten	MN5	[68]	38	Nattenhausen	-	This study
30	Eggingen-Mittelhart3	MN4	[36]	39	Petersbuch2	MN4	[25,26,35,103], this study
31	Erkertshofen2	MN4	[25,26,35,103]	40	Reisensburg	MN4b	[66]
32	Griesbeckerzell	MN6	[25,26,35,103,104]	41	Riedern (Graupensandrinne)	MN4	[104], this study
33	Hambach6	MN5	[27,72]	42	Stätzling	MN5	this study
34	Hohenraunau	MN6	[75]	43	Undorf (Regensburg)	MN4/5	[10]
35	Langenau1	MN4b	[36]	44	Walda2	middle MN5	[35,76,104]
36	Mößkirch	MN5?	[66]	45	Viehhausen	MN5	[10]
	Switzerland						
46	Benken	MN3/4	[65,105]	50	Käpfnach	MN5	[19,106]
47	Brandholz	t.MN5 /b.MN6	D. Kälin comm. pers.	51	Mettlen3	MN5	this study
48	Elgg	MN5	this study	52	Wildensbuch	MN3/4	this study
49	Forenirchel2	MN5	this study				

* is the type locality (in France). References are for the mention of *Amphimoschus* recorded from the localities and/or the age of the locality.

During the last 150 years, the phylogenetical position of *Amphimoschus* has been widely discussed. Bourgeois [4] considered *Amphimoschus* close to the extant musk deer *Moschus*. Subsequently, the genus was successively assigned to the enigmatic and extinct Hoplitomerycidae [5,20,21], included in the Cervoidea [22], or Cervidae [9,15,18,19,23], Bovoidea [17,24–28], "near the origin of bovids" [29], within the Bovidae [6,10,16,30–34], or Ruminantia indet. [35]. Due to the previous lack of information (except for the dentition), the phylogenetic affinities of *Amphimoschus* have remained uncertain and unstable.

Here we review some undescribed cranial remains attributed to *Amphimoschus*, and almost all of the dental material ascribed to this genus in Europe. Therefore, we provide the first detailed and reliable definition of the genus, and we test its phylogenetic affinities with extant and extinct ruminant taxa.

The skull remains have been discovered in France (Artenay, Sable de l’Orléanais Formation), Germany (Langenau1, Obere Brackwassermolasse Gruppe), and Switzerland (Wildensbuch and Benken, Obere Meeresmolasse Gruppe). The exceptional preservation of the basicranial and ear regions adds important insights to the phylogenetic issue. Moreover, the study of 780 teeth provides critical data about the variability of *Amphimoschus* and the validity of the species. Finally, a complete ontogenetic series, comprising neonate, juvenile, adult, and aged stage, is described for the first time.

## Material and methods

### Material

The studied specimens come only from Western and Central European localities: France (Aérotrain, Artenay, Beaugency, Chevilly, Chilleurs-aux-Bois, Faluns d’Anjou, Montréal du Gers, Pellécahus, Pontlevoy, Tavers, Thenay), Germany (Burg-Balzhausen, Erkertshofen 2, Griesbeckerzell 1, Hohenraunau, Langenau1, Mutterhofen, Nattenhausen, Petersbuch 2, Stätzling, Reisensburg, Undorf, Viehhausen, Walda 2), and Switzerland (Benken, Brandholz, Forenirchel 2, Kapfnäch, Mettlen 3 Wildensbuch). Size data from the Eggingen-Mittelhart specimens had been provided by Sach and Heizmann [36]. Unfortunately, specimens studied by our deceased colleague (Léonard Ginsburg) are not described in detail in his contributions. The other fossils are housed at the Naturmuseum Augsburg, Germany (NMA), Naturhistorisches Museum Basel, Switzerland (NMB), the Paläontologische Museum der Universität Zürich, Switzerland (PIMUZ), the Musée d’Histoire Naturelle Toulouse, France (MHNT), the Museum National d’Histoire Naturelle de Paris, France (MNHN), the Musée des Sciences Naturelles d’Orléans, France (MSNO), the Staatliche Naturwissenschaftliche Sammlungen Bayerns—Bayerische Staatssammlung für Paläontologie und Geologie, Germany (SNSB-BSPG), the Staatliches Museum für Naturkunde Stuttgart, Germany (SMNS), and from the private collection of Dr. D. Kälin. “*No permits were required for the described study*, *which complied with all relevant regulations*.” All measurements were taken with a precision of 0.1 mm. 3D models of the skull MNHN.F.Ar3466 (based on a scanning of the surface) and the ear regions of the skulls MNHN.F.Ar3466 and SMNS 40693 (based on a CT scanner data) are available online [37].

### Dental nomenclature and abbreviations

Dental terminology follows Bärmann and Rössner ([38]; see Fig 2). Cranial and postcranial features are defined in Barone [39]. di, deciduous lower incisor; c, lower canine; d, deciduous lower premolar; p, lower premolar; m, lower molar; C, upper canine; D, deciduous upper premolar; P, upper premolar; M, upper molar.

### Synonymy list

Conventional indications used before the year in the synonymy list follow Matthews [40]: *, the work validates the species; v, the authors have seen the original material of the reference; non, the reference actually does not belong to the species under discussion; p.p., in part; no indications, the authors were unable to check the validity of the reference.

### Statistics

We performed an analysis of variance (ANOVA) to test if the size of the specimens from the various biozones were significantly different, as suggested in previous articles. Then we separated specimens from MN3 and MN4 (representing the supposed smaller species *Amphimoschus artenensis*), and those from MN5 and MN6 (representing the supposed larger species *Amphimoschus ponteleviensis*). The specimens from the Falun Formation were not included in this analysis because they may come from different biozones due to reworking [4,41,42]. The analysis was run on the lower third molar since it is the most diagnostic tooth of the genus.

#### Phylogenetic analysis

Phylogenetic analyses were based on a data matrix encompassing 65 characters (dentition, cranium, and postcrania), and 19 taxa. This matrix is based on the work of Aiglstorfer et al. [43], Sánchez et al. [44], Heckberg [45], and new characters were added (see S1 Data). Several characters were constrained as ordered irreversible (0→1): 12, 29–35, 44–45, and 64. The remaining characters were treated as unordered. Maximum parsimony analyses of this updated character-taxon matrix were performed with PAUP version 4.0a [46] using a heuristic search with 10 000 replicates and 100 trees saved by replication, and ACCTRAN character state optimization. Recent molecular-based phylogenetics suggest that Antilocapridae and Giraffidae are the most basal families of living Pecora, while the Bovidae and Moschidae form a clade which is a sister group to the Cervidae (e.g. [47–49]). However, no morphologically-based analysis has so far produced similar results mostly because of strong homoplasy in all these lineages (for alternative topologies see [44,50,51]). In order to take into account the molecular phylogenies, we applied a topological constraint to our phylogenetic analysis with a molecular backbone of the living species included in this study (*Antilocapra americana*, *Giraffa camelopardis*, *Muntiacus muntiac*, *Cervus elaphus*, *Capreolus capreolus*, *Boselaphus tragocamelus*, *Eudorcas rufifrons*, *Moschus moschiferus*) based on the phylogeny provided by Hassanin et al. [47]. The tragulids *Hyemoschus aquaticus* and the Miocene *Dorcatherium crassum* were used as non-pecoran outgroups. The position of the early Miocene species *Procervulus dichotomus* has been constrained as a stem Cervidae as it possesses antlers, the main synapomorphy of cervids [16,32,52], and it has been widely considered as the oldest known Cervidae [5,10,25,32,43,45,52–56]. Setting this taxon at the base of cervids may help orientate the polarity of the characters in early Miocene pecorans. The first ascertained bovids (*Eotragus* and *Namacerus*), the moschid *Micromeryx*, as well as the extinct antilocaprid *Stockoceros*, are also included in the analysis as well as the early Miocene ruminants *Amphitragulus* and *Dremotherium* as members of the early Miocene radiation of the Pecora without a well-defined family attribution [2], and *Ampelomeryx* as an early member of the giraffomorph Palaeomerycidae [2].

## Sytematic paleontology

Order ARTIODACTYLA Owen 1848 [57]

Suborder RUMINANTIA Scopoli, 1777 [58]

Infraorder PECORA Linnaeus, 1758 [59]

Family UNKNOWN

Genus *AMPHIMOSCHUS* Bourgeois, 1873 [4]

*Type species*. *Amphimoschus ponteleviensis* Bourgeois, 1873 [4].

### New diagnosis

Pecoran ruminant possessing enlarged upper canines; a high and strong, obliquely arranged ectostylid on lower molars without external postprotocristid; and the third lower molar possessing a very well developed entoconulid forming a double crescent or lobe with the hypoconulid. The mesostyles of the upper molars, in labial view, and the metastylids of the lower molars, in lingual view, are inclined anteriorly. The tooth enamel is wrinkled. The tooth crowns are high in comparison to all known contemporaneous European ruminants. The diastema between the upper/lower canine and the upper/lower cheek teeth is almost equal to the antero-distal length of P/p2-4. The maxillary interalveolar crest is concave in ventral view. The cranium and the mandible are massive. The maxillary possesses a strong facial crest at the level of the orbits. The auditory bullae are elongate with an elongate neck, a posterior styloid process, and an anteriorly placed lamina vaginalis. *Amphimoschus* has two lacrimal orifices, and no external lacrimal fossa. The bony labyrinth shows an advanced state of the cochlea, elongated in comparison with contemporaneous ruminants (2.75 turns), detached from the vestibule, and a relatively similar thickness of the different turns. In adults, the petrosal bone has a large epitympanic wing that encircles the promontorium. The apex is blunt. The fossa for the tensor tympani muscle is large and round. The basicapsular groove is present and dorsal.

### Etymology

Genus name means close to the genus *Moschus* in Greek.

### Distribution

Late early Miocene to middle Miocene (late Burdigalian to late Langhian, late Ottnangian to early Badenian, Orleanian to earliest Astaracian, end of MN3 to beginning of MN6) of Eurasia.

*AMPHIMOSCHUS PONTELEVIENSIS* Bourgeois, 1873 [4]

(Figs 3–11, S2 and S3 Data, [37])

v 1838 *C*. *lunatus*; von Meyer, p. 413. [19]

v 1839 *Cervus lunatus* H.v.M.; von Meyer, p. 3. [60]

**Fig 3:**
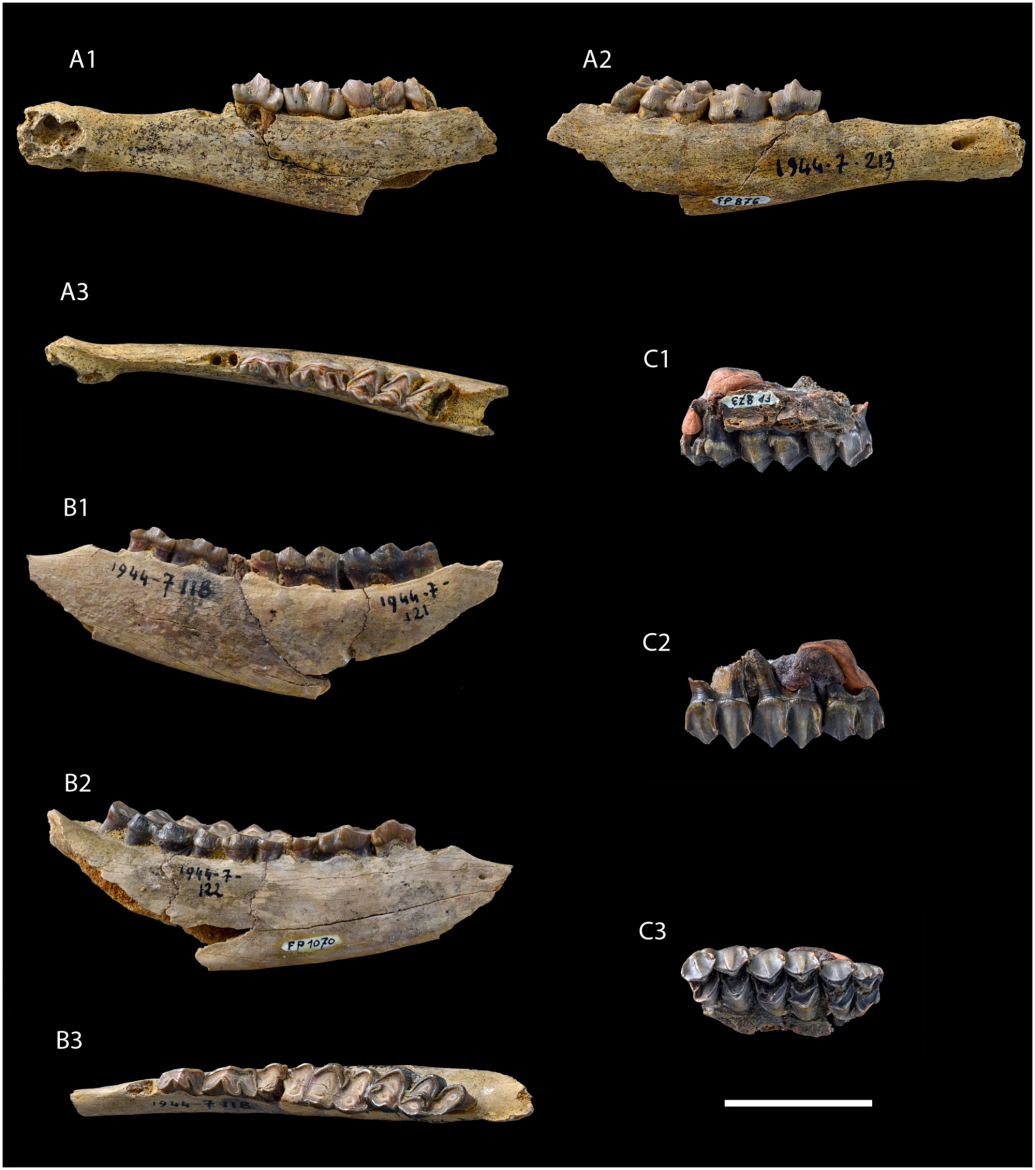
Type series of *Amphimoschus ponteleviensis* (from Pontlevoy, France): Lectotype. **A** MNHN.FP.876, right lower jaw with alveoli of canine, p2, p3-m1, and anterior part of m2's trigonid; Paralectotypes. **B** MNHN.FP.1070, right dental raw with heavily worn p3-m3; **C** MNHN.FP.873, right M1-M3; in lingual, labial, and occlusal views. Scale bar is 2cm.

**Fig 4:**
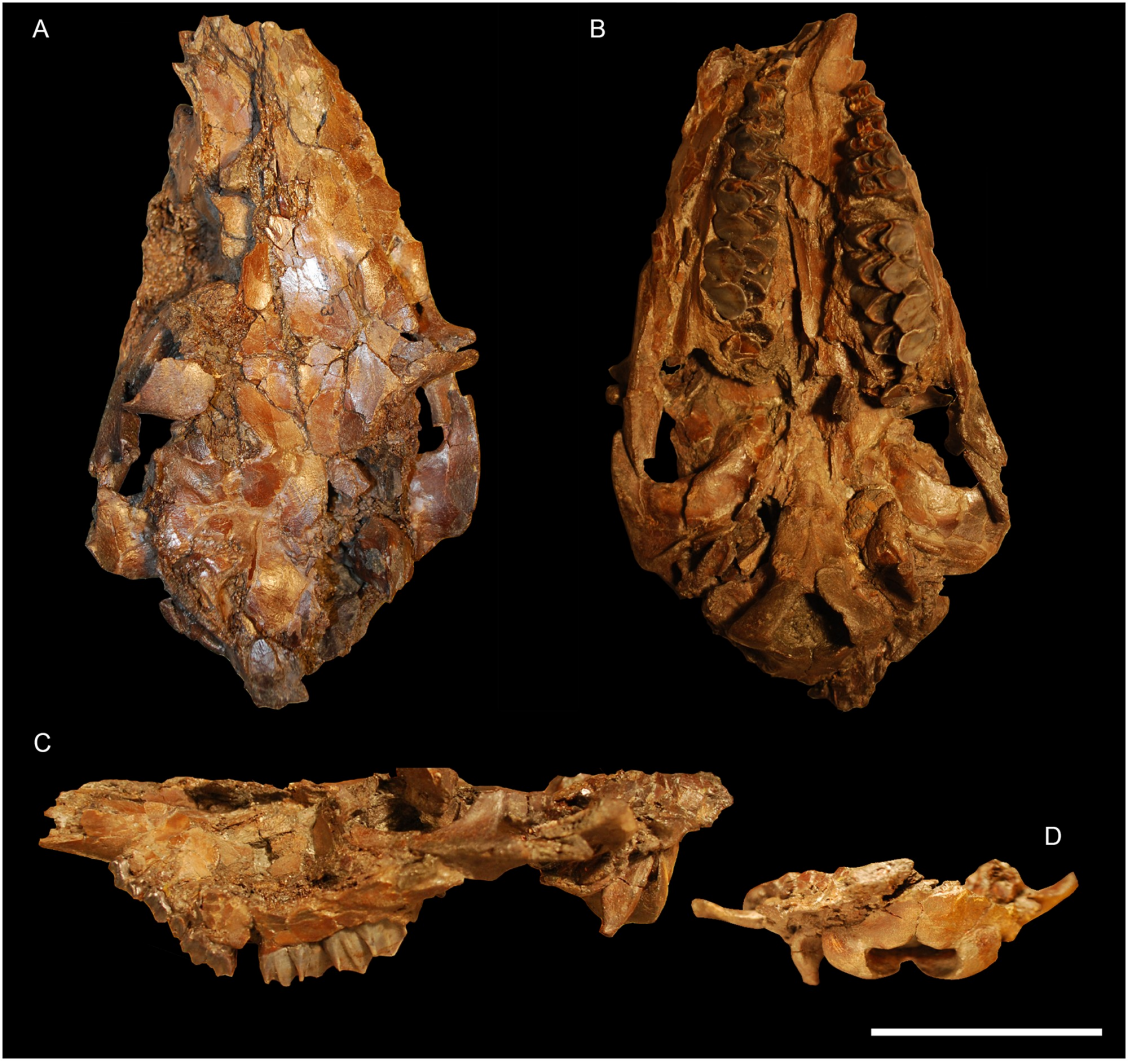
Skull of *Amphimoschus ponteleviensis* from Langenau 1 (Germany). Almost complete juvenile cranium (SMNS 40693) in **A** dorsal view, **B** ventral view, **C** left lateral view, and **D** occipital view. Scale bar is 5cm.

**Fig 5:**
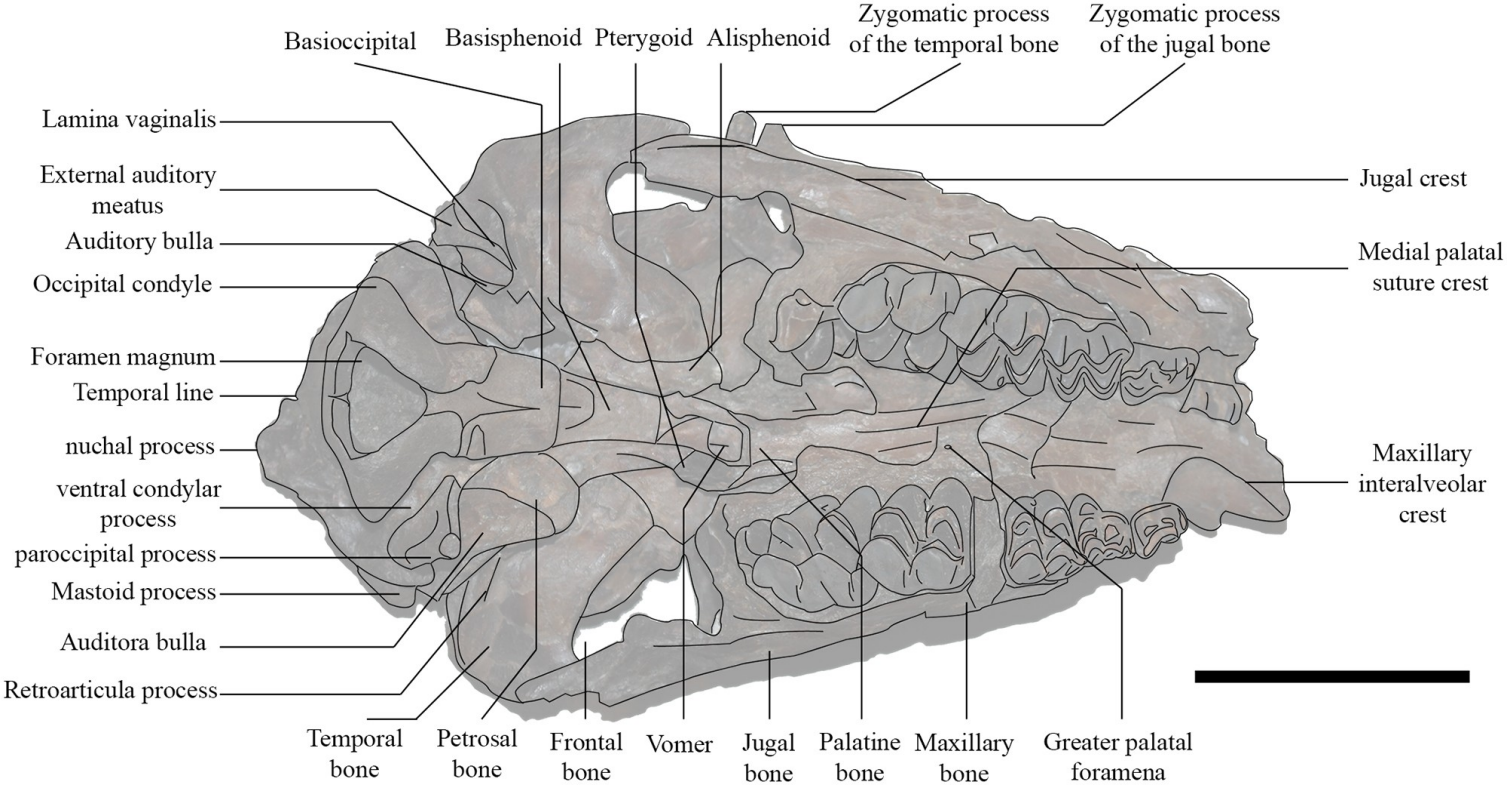
Interpretative drawing of the skull of *Amphimoschus ponteleviensis* from Langenau 1 (Germany) SMNS 40693 in ventral view. Scale bare is 4 cm.

**Fig 6:**
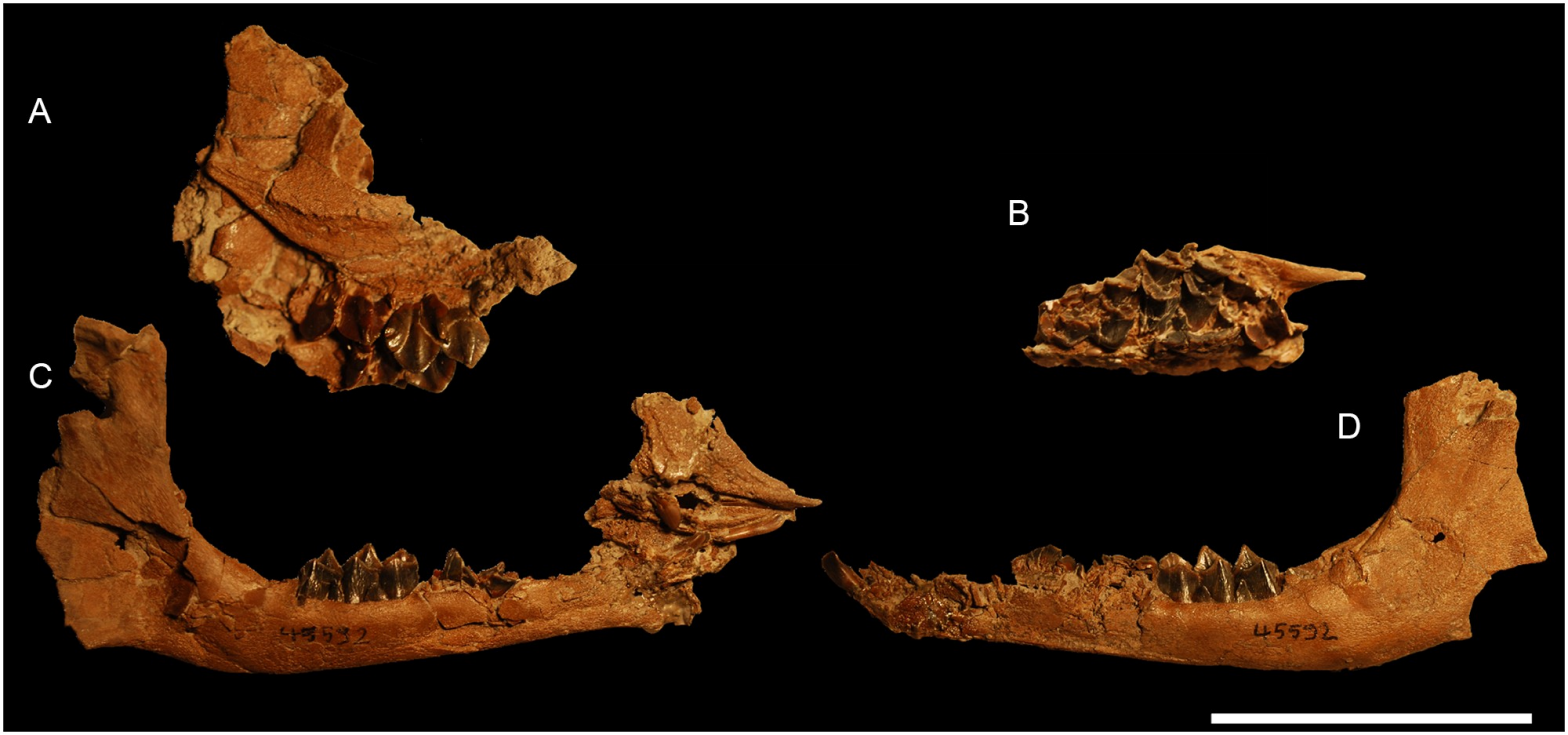
Fragmented skull of *Amphimoschus ponteleviensis* neonate (SMNS 45592) from Langenau 1 (Germany). **A** right maxillary and zygomatic bones with D4-M1 in labial view, **B** left maxillary bone with D3-M1 in occlusal view, **C** right mandible with d4 and incisors, associated with the premaxillary bones, and **D** left mandible with d2-4 and incisor in labial view. Scale bar is 5cm.

* 1873 *Amphimoschus ponteleviensis*; Bourgeois, p. 235, pl. X, Figs 1–6. [4]

v 1887 *Cervus lunatus* H. v. Meyer; Schlosser, pl. V, Figs 14 and 15, pl. VI, Fig 19. [61]

v 1895 *Cervus lunatus Myr*.; Studer, p. 23. [30]

v 1890 *Morphelaphus sansaniensis*; Filhol, p. 262, pl. XXXIX, Figs 4 and 5. [62]

v p.p. 1902 *Antilope cristata* Biederm. (?), “*Cervus lunatus*” v. Mey.; Schlosser, p. 241, pl. IV, Figs 9,10 and 16–18. [63]

v 1907 *Ruminant III*; Stehlin, p. 529. [64]

v 1907? *Ruminant*; Stehlin, p. 535. [64]

v 1908 *Amphimoschus artenensis*, n. sp.; Mayet, p. 141, pl. IV, Fig 16. [9]

v 1908 *Amphimoschus pontileviensis* Bourgeois; Mayet, p. 285, Figs 92 and 93, pl. X, Fig 5. [9]

v 1914 *Amphimoschus lunatus Myr*.; Stehlin, p. 192. [65]

 1924 *Amphimoschus lunatus* v. M.; Klähn, p. 339 [66]

 1924 *Cervus lunatus* v. M.; Klähn, p. 359 [66]

? 1925 *Cervus lunatus* v. M., Klähn p. 204ff, pl. XIII, Figs 35 and 36. [67]

v 1925 *Amphimoschus pontileviensis* Bourgeois; Stehlin, p. 154. [18]

 1934 *Amphimoschus artenensis* Mayet.; Roman and Viret, p. 51, pl. Vi, Fig 28. [15]

 1936 *“Cervus lunatus”* v. M.; Wappenschmitt, p. 53. [68]

v 1956 cf. *Amphimoschus arteniensis* (Mayet, 1908); Rinnert, p. 22ff, pl. 2, Figs 1–3. [10]

v 1959 *Amphimoschus artenensis*; Ginsburg, p. 1. [69]

v 1982 *Amphimoschus artenensis* Mayet; Ginsburg *et al*., p. 404. [70]

v 1993 *Amphimoschus artenensis*; Bulot and Ginsburg, p. 74. [71]

 1993 *Amphimoschus pontileviensis*; Fejfar and Kvacek, p. 17. [11]

v 1997 *Amphimoschus* aff. *artenensis*; Rössner; p. 611ff [20]

v 1997 *Amphimoschus artenensis*; Rössner; p. 611ff [20]

 1999 *Amphimoschus pontileviensis* Bourgeois, 1873; Gentry *et al*., p. 255, Tab. 23.1. [32]

 1999 *Amphimoschus artenensis* Mayet, 1908; Gentry et al., p. 256, Tab. 23.1. [32]

v 2001 *Amphimoschus pontileviensis* Bourgeois 1873; Sach and Heizman, p. 36, pl. 5 Figs 1–5. [36]

v 2001 *Amphimoschus ponteleviensis*; Ginsburg, p. 384, 386, 388–393. [41]

v 2001 *Amphimoschus ponteleviensis* Bourgeois 1873; Rössner and Mörs, p. 594. [72]

non 2003a *Amphimoschus* cf. *A*. *artenensis*; Wang *et al*., p. 262. [13]

non 2003b *Amphimoschus* cf. *A*. *artenensis*; Wang *et al*., p. 74. [14]

non 2004 *Amphimoschus* cf. *A*. *artenensis*; Wang *et al*., p. 411. [73]

v 2004 *Amphimoschus ponteleviensis*; Rössner, p. 94. [26]

v 2004 *Amphimoschus atenensis*; Rössner, p. 94. [26]

 2007 *Amphimoschus pontileviensis*; Cernansky, p. 16. [74]

v 2007 *Amphimoschus artenensis*; Kaiser and Rössner, Tab.1. [35]

v p.p. 2007 *Amphimoschus ponteleviensis*; Kaiser and Rössner, Tab.1. [35]

v 2008 *Amphimoschus ponteleviensis* Bourgeois, 1873; Seehuber, p. 194, pl. 21 Figs 4–8. [75]

v 2010 *Amphimoschus ponteleviensis*; Rössner, p. 152. [76]

v 2011 *Amphimoschus* sp.; Costeur, p. 106. [77]

v 2012 *Amphimoschus pontiliviensis*; Mennecart *et al*., p. 207. [6]

 2012 *Amphimoschus pontileviensis* Gagnaison *et al*., p.230-231, Fig 11. [78]

v 2013 *Amphimoschus artenensis*; Scherler *et al*., ESM2. [2]

v 2013 *Amphimoschus ponteleviensis*; Scherler *et al*., ESM2. [2]

v 2020 *Amphimoschus ponteleviensis*; Mennecart *et al*., Fig 1., 3D data [37]

**Fig 7:**
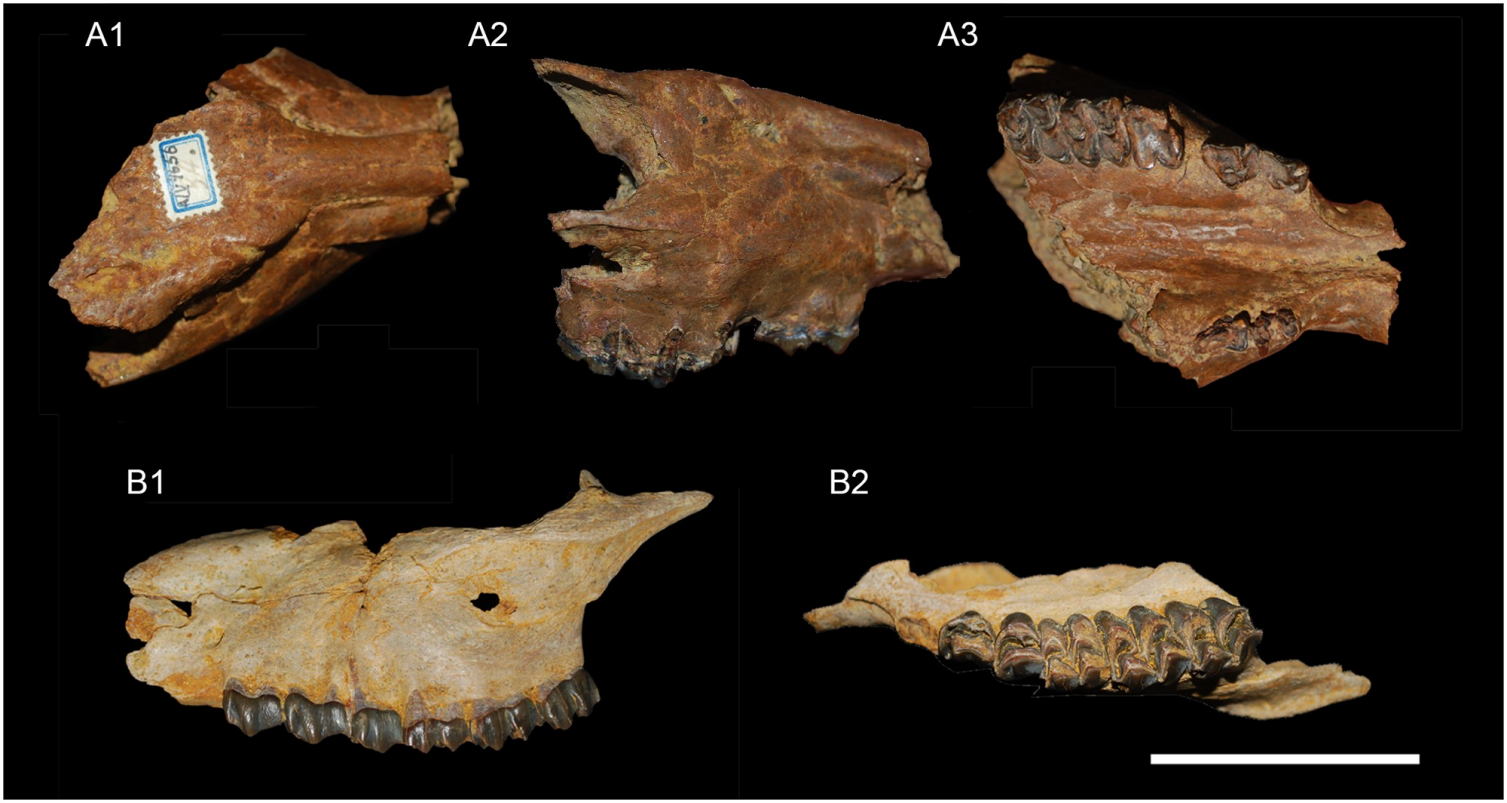
Skulls of *Amphimoschus ponteleviensis* from Wildensbuch and Benken (Switzerland). Wildensbuch: **A** anterior part of the cranium of an elderly individual (PIMUZ A/V4657) in 1 dorsal view, 2 right lateral view, and 3 ventral view; Benken: **B** fragmented cranium (PIMUZ A/V1783) in 1 left lateral view and 2 occlusal view. Scale bar is 5cm.

**Fig 8:**
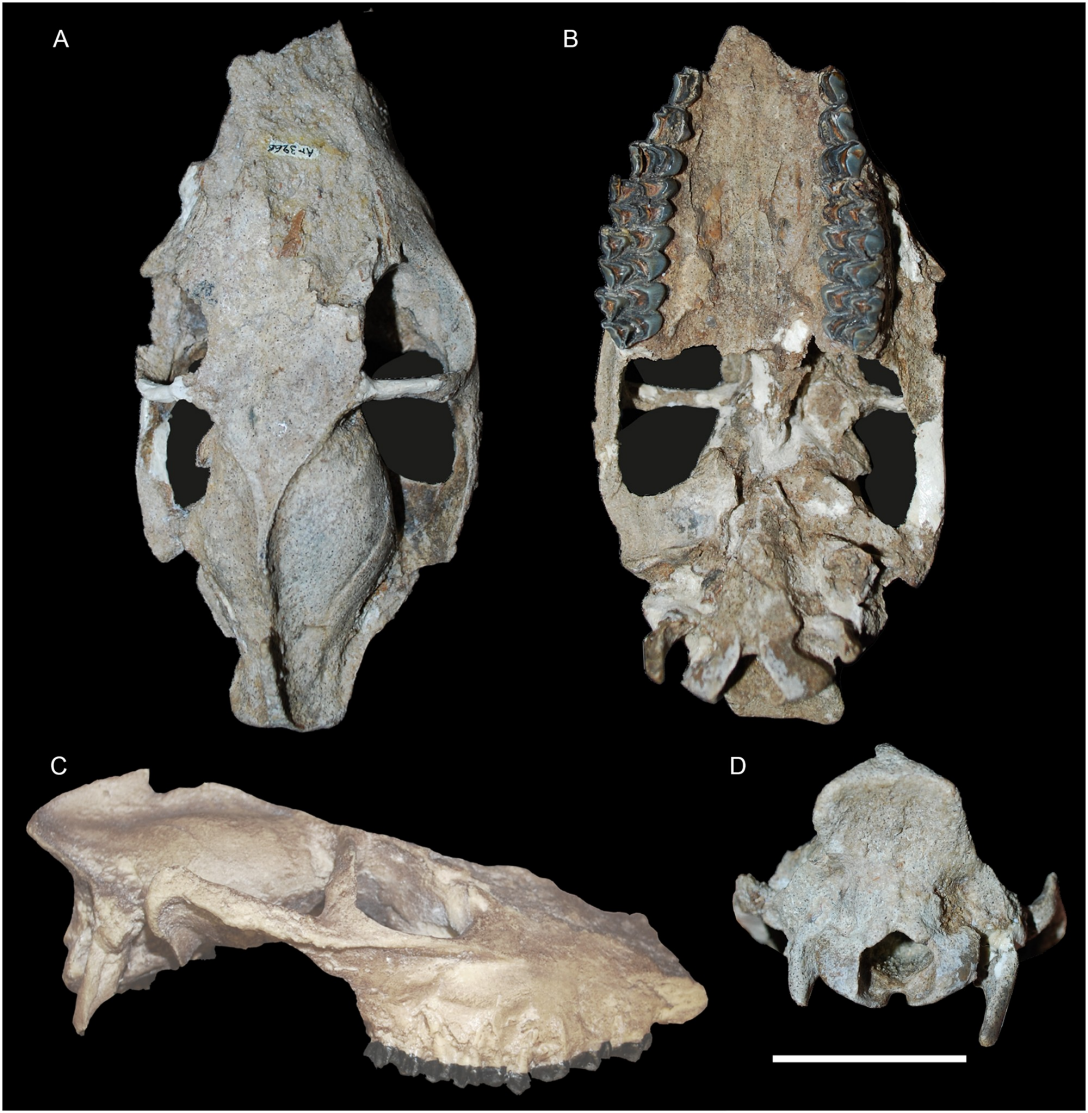
Skulls of *Amphimoschus ponteleviensis* from Artenay (France): Almost complete adult cranium (MNHN.F.Ar3466) in A dorsal view, B ventral view, and C right lateral view, and 4 occipital view. Scale bar is 5cm. (right lateral view provided from the cast of the specimen, 3D model of the cast may be found in Mennecart et al. [37]).

**Fig 9:**
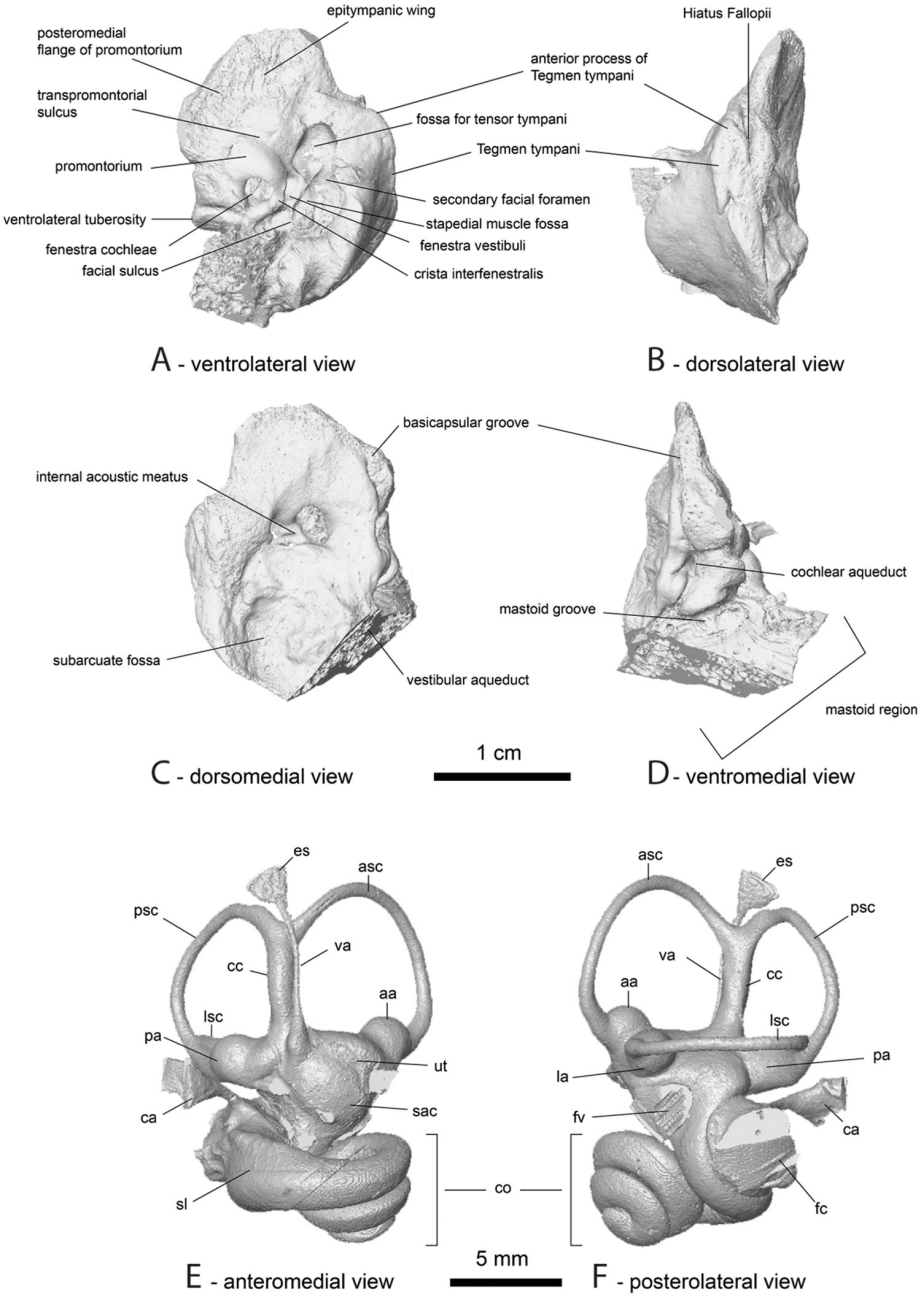
*Amphimoschus ponteleviensis* MNHN.F.AR3266. Petrosal bone in ventrolateral (a), dorsolateral (b), dorsomedial (c) and ventromedial (d) views and bony labyrinth in anteromedial (e) and posterolateral (f) views. aa, asc ampulla; asc, anterior semicircular canal; ca, cochlear aqueduct; cc, common crus; co, cochlea; es, endolymphatic sac; fc, fenestra cochleae; fv, fenestra vestibuli; la, lsc ampulla; lsc, lateral semicircular canal; pa, psc ampulla; psc, posterior semicircular canal; sac, saccule; sl, secondary lamina; ut, utricule; va, vestibular aqueduct. (3D model of the petrosal bones and bony labyrinths of MNHN.F.AR3266 and SMNS 40693 may be found in Mennecart et al. [37]).

**Fig 10:**
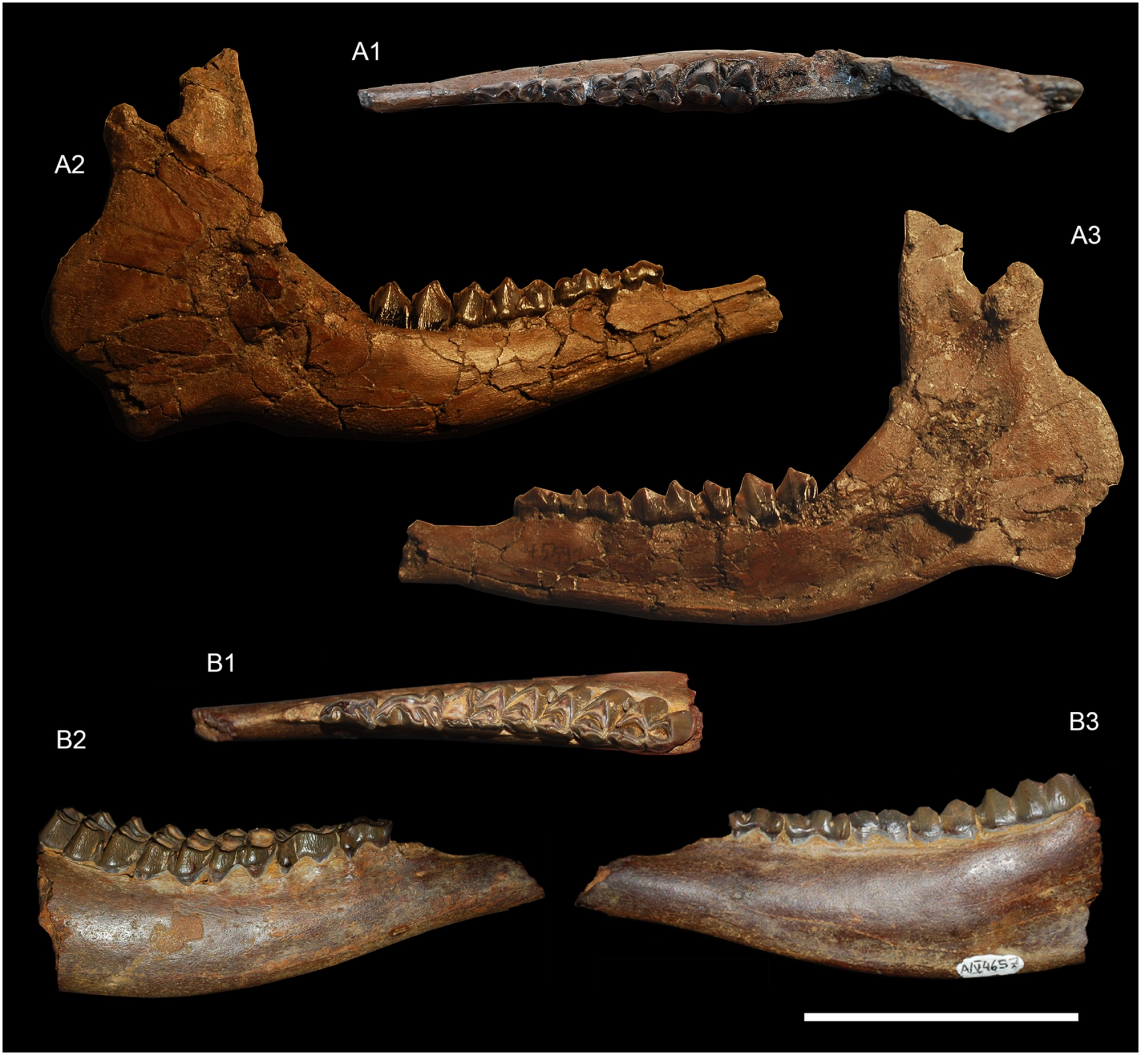
*Amphimoschus ponteleviensis* mandibular remains from Langenau 1 (Germany) and Benken (Switzerland) respectively. Langenau 1: **A**, right mandible with d2-m1 (SMNS 45591), 1 occlusal view, 2 lingual view, and 3 labial view; Benken: **B** right mandible with p3-m3 (PIMUZ A/V4657), 1 occlusal view, 2 lingual view, and 3 labial view. Scale bar is 5cm.

**Fig 11:**
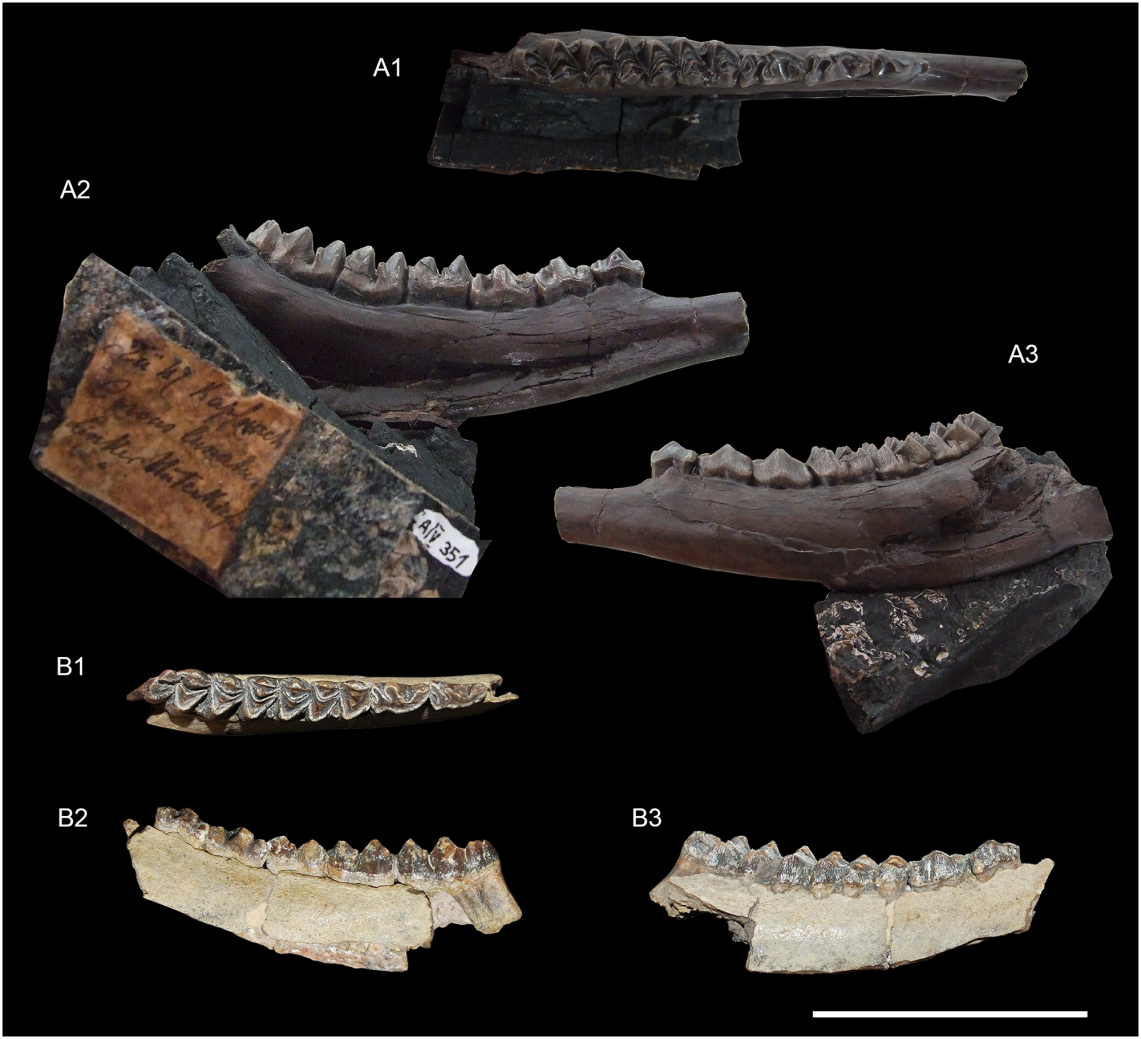
*Amphimoschus ponteleviensis* mandibular remains from Käpfnach (Switzerland) and Artenay (France) respectively. Käpfnach: **A**, left mandible with p2-m3 (PIMUZ A/V351), 1 occlusal view, 2 lingual view, and 3 labial view; Artenay: **B** right mandible with p3-m3 (MSNO 294, type specimen for “*Amphimoschus artenensis*”), 1 occlusal view, 2 lingual view, and 3 labial view. Scale bar is 5cm.

### Nomenclatural acts

In 1838, von Meyer [19] created the species *Cervus lunatus* from Käpfnach (MN5). However, he did not designate a type specimen, neither did he publish an illustration, nor make a description. He only stated that it cannot be distinguished from the present day deer *Cervus*. Actually, some of the specimens designated as *Cervus lunatus* indeed belong to Cervidae (PIMUZ A/V0334, PIMUZ A/V0349, and PIMUZ A/V0364). Following this, Stehlin [18] synonymized this species with *Amphimoschus pontileviensis* Bourgeois (with the typo). In its original publication, Bourgeois [4] did not designate a type specimen. Four dental remains and fragments of both a humerus and metapodials found in Thenay were illustrated. Stehlin [18] considered that only the three illustrated mandibles should be types without any specific designation. Three of these illustrated specimens were recovered in the collection of the Muséum national d’Histoire naturelle in Paris (MNHN.FP.1070, MNHN.FP.876, and MNHN.FP.873). Here, we erect as lectotype the specimen MNHN.FP.876 figured by Bourgeois (1878, Pl. X, Fig 3 [4]) and Mayet (1908, Fig 92 [9]) since this portion of mandible presents diagnostic characters for the genus “*Amphimoschus*”:the elongated diastema between c and p2, the p4 without a developed mesolingual conid, and a molar with a prominent ectostylid, without an external postprotocristid, a closed posterior basin, and a prehypocristid that does not fuse with any other cristid. Consequently, MNHN.FP.1070 (Bourgeois 1878, Pl. X, Fig 1 [4]) and MNHN.FP.873 (Bourgeois 1878, Pl. X, Fig 4 [4] and Mayet 1908, Fig 93 [9]) are considered as paralectotypes, thus completing the diagnosis of the genus, including the shape of the third lobe of the m3. It is interesting to note that in the original article of Bourgeois [4], the specimens are mentioned as being found in “Pont-Levoy” (= Pontlevoy), while the precise locality is further described later in the text as “Thenay”, a village close to Pontlevoy. Given this, Thenay should be considered as the type locality. Moreover, in this original description of *A*. *ponteleviensis* [4], Bourgeois specifies that the illustrated fossils have been found in the fluvial sands belonging to the Sables de l’Orléanais Formation (MN4) and some reworked specimens can also be discovered in the above-lying layers of the marine deposits from the Falun Formation (MN5b). In Mayet [9], the specimens originally reported by Bourgeois were considered to be found in the Falun-type beds, which served to justify the occurrence of a smaller species, *A*. *artenensis*, in the older locality (MN4) of Artenay. However, the fluviatile beds exposed in Thenay-Pontlevoy are not the Sables de l’Orléanais Formation but the Sables et Marnes du Blésois that are MN5a [8].

### Lectotype

MNHN.FP.876, right lower jaw with alveoli of canine and p2, and tooth row p3-m1, and anterior part of m2's trigonid (figured by Bourgeois 1878, Pl. X, Fig 3; here Fig 3A [4]); size (length x width): p3, 11.3 x 6.4; p4, 13.1 x 7.3; m1, 12.2 x 8.8.

### Paralectotype

MNHN.FP.1070, right lower jaw with heavily worn p3-m3 (figured by Bourgeois 1878, Pl. X, Fig 1; here Fig 3B [4]); size (length x width): p3, 10.1 x 5.9; p4, 12.2 x 6.8; m1 10.7 x 9.1; m2 14.0 x 10.2; m3, 21.5 x 10.1; MNHN.FP.873, right M1-M3 (figured by Bourgeois 1878, Pl. X, Fig 4; here Fig 3C [4]); size (length x width): M1, 13.8 x 13.6; M2, 15.4 x 15.2; M3, 14.5 x 13.5.

### Etymology

The species name refers to the Pontlevoy (Loir-et-Cher, France), the closest city to the Thenay locality where the species was first discovered.

### Type locality and horizon

Thenay, close to Pontlevoy (Loir-et-Cher, France); Sables et Marnes du Blésois Formation MN5a (ca. 16.5–15.5 Ma, Orleanian, late early to early middle Miocene).

### Additional localities and biochronologic range of Amphimoschus ponteleviensis

**CZECH REPUBLIC**: Spolocenstvo; **FRANCE**: Aérotrain (Chevilly), Artenay, Beaugency, Breil, Channay-sur-Lathan, Chavaignes, Chilleurs-aux-Bois, Cléré-les-Pins, Genneteil, Homme, La Romieu, Lasse, Lublé, Meigné-le-Vicomte, Méon, Monréal du Gers, Noyant-sous-le-Lude, Pellecahus, Pont-Boutard, Pontigné, Rillé, Saint Laurent-de-Lin, Savigné-sur-Lathan, Tavers, Thenay, Tourelet; **GERMANY**: Burg-Balzhausen, Eggingen-Mittelhart 3, Erkertshofen 2, Griesbeckerzell1, Hambach 6, Hohenraunau, Langenau 1, Muttershofen, Nattenhausen, Petersbuch 2, Reisensburg, Stätzling, Undorf, Viehhausen, Walda 2; **SWITZERLAND**: Benken, Brandholz, Elgg, Forenirchel2, Käpfnach, Mettlen3, Wildensbuch; end of MN3 to beginning of MN6 (late Burdigalian to late Langhian, late Ottnangian to early Badenian, Orleanian to earliest Astaracian). Remark: as already noticed in Rössner [76], Kaiser and Rössner [35] mistakenly mentioned *Amphimoschus* in Thannhausen.

### Referred material

***Artenay***: MNHN.F.Ar3266 cranium of a mature individual, MSNO 294 right mandible with p3-m3 (type of *Amphimoschus artenensis*, illustrated in Mayet 1908 Pl. V, Figs 4 and 5), NMB SO71 right fragmentary mandible with worn p4-m3, NMB SO72 right fragmentary mandible with worn m1-m3, NMB SO80 right M1-3, NMB SO187 right p2-m3, NMB SO268 fragmentary mandible with p3-m3, NMB SO3510 right m1-3, NMB SO3511 mandible with m1-3, NMB SO3512 left m1-3, NMB SO3922 right mandible with worn p4-m3, NMB SO4079 well preserved right mandible with p3-m3, NMB SO1303 left m3, NMB SO1304 right m3, NMB SO14072 left mandible with p4-m3, NMB SO1895 right M2, NMB SO2108 right P2, NMB SO2348 left P4-M2, NMB SO2414 left m3, NMB SO2633 right M2, NMB SO2644 right m2, NMB SO271 right m3, NMB SO272 right m2, NMB SO3148 right M2, NMB SO3149 right m2, NMB SO3280 left M2, NMB SO3470 left P2-P4, NMB SO3511 right mandible with p4-m3, NMB SO3514 left M1, NMB SO3515 left m2, NMB SO3516 right m2, NMB SO3518 left m3, NMB SO3531 right m3, NMB SO3532 left m2, NMB SO3556 left mandible with m2-3, NMB SO3557 right m3, NMB SO3923 left m3, NMB SO40 left mandible with p3-4, NMB SO4071 left mandible with m2-3, NMB SO4080 right m3, NMB SO413 left m1, NMB SO437 right M3, NMB SO439 right m3, NMB SO4416 right M2, NMB SO4417 right M2, NMB SO444 left p4, NMB SO463 right p4, NMB SO480 left m2, NMB SO545 right M1,NMB SO5503 left P4-M2, NMB SO5927 right mandible with m2-3, NMB SO5928 right mandible with p3-4, NMB SO6296 left m1, NMB SO6298 left m3, NMB SO64 left m3, NMB SO67 left m1, NMB SO68 right m2, NMB SO69 left m1, NMB SO70 left m1, NMB SO74 right mandible with m2-3, NMB SO75 right mandible with m2-3, NMB SO78 right mandible with d4-m1, NMB SO80 right M1-3, NMB SO831 left m3, NMB SO831 right p4, NMB SO852 left M2, NMB SO861 left mandible with p3-m3, NMB SO87 right M2, NMB SO88 right M1, NMB SO89 left M1, NMB SO90 left M1; ***Benken***: PIMUZ AV 4657 right mandible with p3-m3, PIMUZ A/V4673 right M1-M2, PIMUZ A/V4674 left m3, PIMUZ A/V4675 left P3-M2, PIMUZ A/V4676 left M1-M3, PIMUZ A/V4677 right P3, PIMUZ A/V4678 right M1, PIMUZ A/V4679 right M1, PIMUZ A/V4680 left M2, PIMUZ A/V4681 left M2, PIMUZ A/V4682 right M3, PIMUZ A/V4683 left M3, PIMUZ A/V4684 left m3, PIMUZ A/V4685 left d4, PIMUZ A/V4686 left M1, PIMUZ A/V4687 left m3, PIMUZ A/V4688 right M1, PIMUZ A/V4689 right M1, PIMUZ A/V4690 right M2, PIMUZ A/V4691 left P3, PIMUZ A/V4692 right P2, PIMUZ A/V4693 right p4, PIMUZ A/V4694 left M2; ***Burg-Balzhausen***: NMA 2005/228/1927 left m2, NMA 2005/229/1927 right M3; ***Chevilly/Aérotrain***: NMB SO5714 right M1, NMB SO5713 left m1; ***Chilleurs aux Bois***: NMB 766 left mandible with p3-m3, NMB SO63 left mandible with p3-m3, NMB SO93 left mandible with m1-3, NMB SO2427 left mandible with m1-3, NMB SO476 right m2, NMB SO2589 left m3, NMB SO2590 left m3, NMB SO2335 right m1, NMB SO4306 left mandible with p4-m3, NMB SO188 right mandible with p2-p4, NMB SO6392 right mandible with p3-m3, NMB SO314 left m3, NMB SO2430 left M1, NMB SO310 left mandible with p4-m3; ***Elgg***: NMB OSM12 right m3, Elgg NMB no n° right p3; ***Erkertshofen 2***: SNSB-BSPG 1974 XIV 9907 right M1, SNSB-BSPG 1974 XIV 9909 left M2, SNSB-BSPG 1974 XIV 9911 left M3, SNSB-BSPG 1974 XIV 10149 right P2, SNSB-BSPG 1974 XIV 10154 right P2, SNSB-BSPG 1974 XIV 10341 right p4, SNSB-BSPG 1974 XIV 10242 right D2, SNSB-BSPG 1974 XIV 10968 left D2; ***Forenirchel 2 (Freienstein)***: PIMUZ A/V3158 left D3; ***Griesbeckerzell1***: SNSB-BSPG 1997 XIII a 139 right m2, SNSB-BSPG 1997 XIII a 402 left d4, SNSB-BSPG 1997 XIII a 412 left p4, SNSB-BSPG 1997 XIII a 162 right M1, SNSB-BSPG 1997 XIII a 409 left P4, SNSB-BSPG 1997 XIII b 462 left m1-m3, SNSB-BSPG 1997 XIII a 399 left D2, SNSB-BSPG 1997 XIII a 411 right p3; ***Hohenraunau***: NMA 2005/222/1633 left P2-3 and right M1-3, NMA 2005/225/1927 left M; ***Kapfnäch***: PIMUZ A/V0331 fragmented left m3 and right m2-m3, PIMUZ A/V0332 left m2-m3, PIMUZ A/V0334 right fragmented mandible with p4-m2, PIMUZ A/V0335 left p4-m2, PIMUZ A/V0337 fragmented skull with left P4-M3 and right P3-M1, PIMUZ A/V0338 left maxilla with P2-M3, PIMUZ A/V0339 right p2-m3, PIMUZ A/V0340 right mandible with p3 and m1 and left mandible with p2-p4 and fragmented m2-m3, PIMUZ A/V0342 fragmented right mandible with broken d4 and m1-m2, PIMUZ A/V0343 fragmented right mandible with broken p3 and m2 and complete p4-m1 and left p4-m1, PIMUZ A/V0345 fragmented right mandible with p2-p4, PIMUZ A/V0346 fragmented left mandible m1-m3, PIMUZ AV 46 right mandible with p2-m3 and left m2, PIMUZ A/V0347 fragmented left p3-m1, PIMUZ A/V0348 fragmented right mandible with m1-m3, PIMUZ A/V0350 left fragmentary mandible with m2-3, PIMUZ A/V0351 left mandible with p2-m3, PIMUZ A/V0352 fragmented left mandible with p2-m3, PIMUZ A/V0353 fragmented skull with left and right M1-M3, left mandible with m1-m3 and right m3, PIMUZ A/V0355 vertebra and flattened skull with right P3-M2 and left P2-m3, PIMUZ A/V0356 fragmented skull with right P4-M1 and left M2-M3, PIMUZ A/V0357 fragmented skull with left P2-M3 and right M1-M3, PIMUZ A/V0358 left mandible with p2-m3 and left maxillary with fragmentary p2-m2, PIMUZ A/V0359 left mandible with p4-m3, PIMUZ A/V0360 left mandible with p3-p4 and right mandible with p4-m3, PIMUZ A/V0362 right m2-m3, PIMUZ A/V0363 left M1-M3; ***Langenau1***: SMNS 40693 cranium of a young individual, SMNS 40694 right mandible with broken d4, m1-2, and erupting m3, SMNS 40696 right M1, SMNS 40698 right D4, SMNS40723 metacarpus, SMNS 45591 almost complete right mandible with d2-m1, SMNS 45592 fragmented neonate cranium with mandibles, SMNS 47441 left mandible with p2-4, SMNS 47442left mandible with m1 and fragmentary m2; ***Mettlen 3***: PIMUZ A/V3592 right d4; ***Montréal-du-Gers***: MHNT 1063 left mandible with p4-m3, MHNT Béon F2 824 m2-m3, MHNT Béon 1993 G2 630 m2-m3, MHNT Béon 1991 G2 956 m1-m2, MHNT Béon G2 851 d4-m1, MHNT Béon F2 824 p2-p4, MHNT Béon 89 G2 650 p2-p4, MHNT Béon 1991 P2-M3, MHNT Béon 93 G3 1361 P2, P3, M2, M3; ***Muttershofen***: NMA/1633 right m3; ***Nattenhausen***: NMA/1415 right m1-3 and left m; ***Pellecahus***: NMB GB2467 right p4, NMB GB2465 left M3, NMB GB2464 right M1; ***Petersbuch2***: SNSB-BSPG 1976 XXII 9907 right M1, SNSB-BSPG 1976 XXII 10154 right P2, SNSB-BSPG 1976 XXII 10968 left D2, SNSB-BSPG 1976 XXII 9909 left M2, SNSB-BSPG 1976 XXII 10242 right D2, SNSB-BSPG 1976 XXII 10149 right P2, SNSB-BSPG 1976 XXII 10341 right p4, SNSB-BSPG 1976 XXII 9911 left M3; ***Reisensburg (Günzburg)***: SNSB-BSPG 1881 IX 51 right P3 (illustrated in Schlosser 1887: Taf. V Fig 14 [61]), SNSB-BSPG 1881 IX 668 right P3, SNSB-BSPG 1881 IX 673 left M1/M2 (illustrated in Schlosser 1902: Taf. IV Fig 9 [63]), SNSB-BSPG 1881 IX 669 right P2 (illustrated in Schlosser 1902: Taf. IV Fig 9 [63]), SNSB-BSPG 1881 IX 671 (51d) left p4 (illustrated in Schlosser 1902: Taf. IV Fig 18 [63]), SNSB-BSPG 1881 IX 670 right d4 m1 (illustrated in Schlosser 1887: Taf. V Fig 15 [61]), SNSB-BSPG 1881 IX 672 right d4, SNSB-BSPG 1881 IX 675 right m1, SNSB-BSPG 1881 IX 674 left M1, SNSB-BSPG 1881 IX 667 left P4-M3 (illustrated in Schlosser 1902: Taf. IV Fig 9 [63] and Schlosser 1887: Taf. VI Fig 19 [61]), SNSB-BSPG 1881 IX 666 right p3-m3 (illustrated in Schlosser 1902: Taf. IV Fig 10 [63]); ***Stätzling***: NMA Coll NVS 148 left P3-4; ***Tavers***: SNSB-BSPG 1993 IX 37 right M3, SNSB-BSPG 1993 IX 38 left p4-m3, SNSB-BSPG 1993 IX 39 left p4-m3, SNSB-BSPG 1993 IX 40 left m2-m3, SNSB-BSPG 1993 IX 42 left m3, SNSB-BSPG 1993 IX 41 left m1, SNSB-BSPG 1993 IX 120 right P3; Thenay (close to ***Pontlevoy)***: MNHN.FP.2696 left mandible with p2-m3 (illustrated in Mayet 1908: Fig 92 [9]), MNHN.FP.836 left p4-m3;***Undorf (Regensburg)***: SNSB-BSPG 1935 II 507 left m3 (illustrated in Rinnert 1956: P. 22ff [10]), SNSB-BSPG 1935 II 511 left m1/m2 (illustrated in Rinnert 1956: P. 22ff [10]), SNSB-BSPG 1935 II 510 right p4 (illustrated in Rinnert 1956: P. 22ff [10]); ***Viehhausen (Regensburg)***: SNSB-BSPG 2008 LI 126 right p2-m1 (illustrated in Rinnert 1956: p. 22ff, Taf 2 Fig 1 [10]), SNSB-BSPG 2008 LI 201 left? M3 (illustrated in Rinnert 1956: P. 22ff [10], listed in Rinnert 1956 as Taf. 2 Fig 2, but this is incorrect [10]), SNSB-BSPG 2008 LI 224 left P2 (in Rinnert 1956 left P3 [10]) P4 and? M3 and right P2-P3 (illustrated in Rinnert 1956: p. 22ff, Taf 2 Fig 2 [10]), SNSB-BSPG 2008 LI 343 left m3 (illustrated in Rinnert 1956: P. 22ff [10]), SNSB-BSPG 2008 LI 345 left m3 (illustrated in Rinnert 1956: P. 22ff [10]), SNSB-BSPG 2008 LI 581 left p2-p3 (illustrated in Rinnert 1956: p. 22ff, Taf 2 Fig 1 [10]); ***Walda2***: SNSB-BSPG 1987 V 250 left D2, SNSB-BSPG 1987 V 272 right m2, SNSB-BSPG 1987 V 434 left d3, SNSB-BSPG 1987 V 251 right D2, SNSB-BSPG 1987 V 273 right D3, SNSB-BSPG 1987 V 445 left d3, SNSB-BSPG 1987 V 256 left p2, SNSB-BSPG 1987 V 274 left M3, SNSB-BSPG 1987 V 452 left d3, SNSB-BSPG 1987 V 268 left p4-m2, SNSB-BSPG 1987 V 275 right m1-m3, SNSB-BSPG 1987 V 466 left D3, SNSB-BSPG 1987 V 270 right P2, SNSB-BSPG 1987 V 401 right M2, SNSB-BSPG 1987 V 271 left P3, SNSB-BSPG 1987 V 407 right m3; ***Wildensbuch***: PIMUZ A/V1783 anterior half of a skull, PIMUZ A/V4656 fragmented skull, PIMUZ A/V4658 fragmented mandible with m2-m3, PIMUZ A/V4695 left maxilla with P2-M2, PIMUZ A/V4696 left D4-M1, PIMUZ A/V4697 right M2, PIMUZ A/V4698 left p4, PIMUZ A/V4699 right m2, PIMUZ A/V4700 left M2, PIMUZ A/V4701 left m3, PIMUZ A/V4702 right m2, PIMUZ A/V4703 left m1, PIMUZ A/V4704 left P4-M1, PIMUZ A/V4705 left M1-M3, 4794 fragmented right mandible with m2. All the measurements are given in S2 Data.

### Diagnosis

As for the genus *Amphimoschus*.

## Description

### Skull SMNS 40693 (Figs 4 and 5)

The skull SMNS 40693 is a cranium of a young individual with left and right deciduous tooth rows, M2 has totally emerged and M3 is erupting. The skull suffers strong dorsoventral flattening with a small amount of distortion. This skull is almost complete. Only the premaxillary bones are missing. The upper part of the skull is broken into small pieces. It is very hard to identify the bone sutures. The right basilar area is the best-preserved part of the hornless skull.

#### Facial region

The maxillary bones are preserved from the anterior part of the left diastema and the right D2. The left canine is partially erupted. Only its elongated root is partially preserved on the skull. Its apex is separated from the skull and not worn at all. The maxillary bulge above the left canine root makes a groove on its upper part separating the maxilla from the nasal bone. The nasal bones are broken along their anteroposterior length. The lacrimal foramina and the infraorbital foramina are not visible due to the poor preservation of the specimen in that area. There is no lacrimal fossa. The maxillary interalveolar crest is preserved on the left side of the skull. It is highly concave and slightly deformed by distortion. It joins the anterior part of D4. The left greater palatal foramen is located at the level of M1. The medial palatal suture is quite flat, with a small crest in the middle.

#### Orbital region

Both orbits are complete, but are flattened and disarticulated. The orbits are quadrangular with a flat upper and lower surface. Both zygomatic arches are very strong and bear a huge facial crest. Both zygomatic processes of the frontal bones are preserved, but dislocated. The frontal processes of the zygomatic bones are tilted slightly backwardly. The dorsal part of the frontal bones is fragmented. Both supraorbital foramina are preserved with a large supraorbital groove. Behind the orbits, the frontal bones possess an oblique temporal line, which is linked to the sagittal crest. The lacrimal bones cannot be distinguished. This fossil differs from PIMUZ A/V4656 (Fig 7A) in lacking the facial crest above the orbits.

#### Dorsocranial-temporal region

The left parietal bone is fragmentary. The right temporal bone is the best preserved. The retroarticular process is well developed. It lines the anterior part of the auditory bullae, just at the level of the external auditory meatus. The alisphenoid wing, which is just in the axis of the retroarticular process, forms another crest. The posterior part of the temporal bone is poorly preserved. Only the left mastoid process is preserved. Its crest joins the well-developed nuchal crest. The mandibular fossa is shallow. The zygomatic process of the temporal bone exhibits a small tuberosity on its ventral part.

#### Nuchal region

The occipital bone is well preserved. The nuchal crest is well developed. The protuberance formed by this crest is prominent. The nuchal tuberosity is cracked on its upper part. The foramen magnum is circular. The occipital condyles are triangular in caudal and ventral views. They extend by a rather large tuberosity. On the basilar part of the occipital bone, there is a spine protruding from the axis of the bone. The hypoglossal nerve canals are both preserved. They are large and oval, positioned at the level of the basilar bend. They cross the occipital condyles and can be observed on the proximal part of the ventral condylar fossa. Another foramen, probably the vascular foramen, is situated at the end of the vascular fossa.

#### Basicranial region

The basioccipital bone is rectangular in shape. The left paroccipital process is well preserved. It is triangular in shape in posterior view, flat ventrally, rounded dorsally. Close to the suture with the basisphenoid bone, there are the muscular tuberosities of the basilar process. The basisphenoid bone is short. The pterygoid process borders it. The left pterygoid hamulus is preserved. On the alisphenoid bone, there is a small oval foramen just between the styloid process and the basisphenoid bone. The palatine bones are preserved but cannot be distinguished from maxillary bones. No foramen can be observed. The choanae are preserved in three dimensions and rectangular in shape. The start of the vomer is located at the middle part of the choanae.

#### Auditory region

The left auditory bulla is almost in anatomical position. The right bulla is broken and the petrosal bone has been moved to the top of the skull roof, through the frontal bones. The left external auditory meatus has a partially preserved vaginal process and is broken. The right external auditory meatus is well preserved. It is circular, with a notch on its ventral part. The left part of the skull exhibits the mastoid process, the external auditory meatus, the retroarticular process and the mandibular fossa. The two petrosal bones can be seen on the dorsal part of the skull. They are triangular in section due to their insertion with the occipital bone just below the ventral condylar process. The petrosal crest can be observed on the dorsal part of the skull. The petrosal bones are described later in detail. The caudal border of the left bulla is at the level of the rostral border of the basioccipital bone. On its posterior part, the bulla touches the paroccipital process, forming a narrow and triangular depression between the bulla and the basioccipital. The external auditory meatus is elongate, straight, and ended by the bulla, which is oriented at an acute angle. The bullae are bean-shaped (larger dorsoventral dimension than rostrocaudal) and have a smooth surface. The right bulla is slightly compressed anteriorly. The right vaginal lamina extends to the middle of the bulla. Due to poor preservation, the styloid process cannot be seen, but it is located on the posterior part of the bulla. The caudal ends of both bullae are tapered and the muscular processes are lacking. The left external auditory meatus is circular in section with two notches. The vaginal process begins on the median part of the external auditory meatus orifice and runs rostrally, enclosing a groove between the auditory bulla and itself. This groove cannot be seen on the left side, because the bulla is flattened towards the vaginal process.

### Skull SMNS 45592 (Fig 6)

The specimen SMNS 45592 belongs to a young individual. The first molars are erupting. The mandibles are well preserved with erupting D3-4. The left one also possesses an I3 and D2 in place. The right premaxilla is associated with the right mandible. It is toothless, high and triangular in lateral view. In inner view, the palatal fissure is elongated, narrow like the palatal process, and rounded at its extremity. The premaxillary bones are elongated and slightly enlarged on their rostral part. The nasal cavity is wide and separated in two parts by a fragmented ethmoidal bone. The left maxillary bone has D3-4 associated with the zygomatic bone, which has a facial crest. The right maxillary bone has a D4 and an erupting M1. Other skull bones are associated to this specimen: fragments of the frontal bone, the zygomatic bone with the frontal process, the temporal process, the orbital face and a very strong zygomatic crest.

### Skull PIMUZ A/V4656 (Fig 7A)

PIMUZ A/V4656 is a cranium of an aged individual with full postcanine dentition except left and right P4, which are missing (pathological condition). The crowns of the teeth that are present are highly worn. The caudal part of the skull is missing, i.e. broken transversally, from rostral of the left orbit and at the level of the missing P4 to the middle part of the right orbit and caudal of the right M3. This fossil is preserved in 3D, with a small deformation of the right maxillary bone. This skull is robust and globular.

#### Facial region

The premaxillary bones are missing. The maxillary bones are preserved from the caudal edge of the canine alveolus opening. The alveolus of the enlarged upper canine runs in an arch along the dorsal edge of the rostral maxilla. The nasal bones are missing their rostral elongation. In rostral view, they are long, regularly laterally curved, relatively narrow, and straight. Their contact with frontal bones is straight. The infraorbital foramens are large and oval. There is no lacrimal fossa. The maxillar interalveolar crest on the diastema between canine and P2 is preserved on both sides of the skull. It is strongly arched towards medial, such as in *Bos taurus* Linnaeus, 1758, and the extinct *Bedenomeryx* Jehenne, 1988 [79], which differs from the extinct *Micromeryx* Lartet, 1851 [80] (e.g. [43]) and the living *Moschus* Linnaeus, 1758 [59] where the crest is straight and parallel to the sagittal plane. The greater palatal foramina are small, located at the level of the posterior part of the M1, with a clear groove just rostral of it running up to the level of P3. In ventral view, the palate is transversally concavely arched, with a weak medial palate suture crest formed by the suture of these bones.

#### Orbital region

Only the right orbit is partially preserved. The ventral and dorsal portions of the orbit are flat. The frontal bones are well preserved in the area of the articulation with nasal and lacrimal bones. In lateral view, their dorsal profile is flat as is the case with the nasal bones. There is a prominent supraorbital ridge from the nasal bone forming the dorsal edge of the orbit. At the rostral end of this crest, a sigmoid supraorbital groove joins a large supraorbital foramen. There is the typical double lacrimal orifice of cervoids, in dorsoventral arrangement. The lacrimal bones are flat and arranged in a sub-sagittal plane. The lacrimal bones do not to reach the nasal bones due to the presence of a small antorbital vacuity and the lack of a caudal lacrimal process. The facial crest is well marked below the orbit.

### Skull PIMUZ A/V1783 (Fig 7B)

PIMUZ A/V1783 consists of two fragments of skull belonging to a young adult. All the teeth are little worn. Only the left and right maxillary and zygomatic bones are partially preserved. All permanent teeth are erupted; the right P2 is missing.

#### Orbital region

The facial crest begins at the level of the orbits and continues on the huge and sigmoidal zygomatic arch. The oval infraorbital foramen is large and dorsoventrally elongated. Its external opening is located directly rostral, just in front of P2 and internally it opens between P2 and P3. The bulge running along the rostro-dorsal edge of the maxillary bones houses the canine root and its alveolus. The interalveolar crest between C and P2 is partially preserved and arched towards medial in palatal view. The maxillary bone does not extend caudally beyond M3.

### Skull MNHN.F.Ar3266 (Fig 8)

The cranium MNHN.F.Ar3266 is an almost complete skull. Similarly to PIMUZ A/V1783, all of the teeth are little worn. Only its rostral part and auditory region are damaged. The bone sutures are fully fused and cannot be traced because of both the age of the individual and the preservation.

#### Orbital region

Both orbits are quadrangular with flat dorsal, ventral, and caudal edges. Both zygomatic arches are very strong and possess a huge facial crest. The frontal processes of the zygomatic bones are perpendicularly arranged. The left supraorbital foramen is preserved with a small and weak supraorbital gulley. Caudal to the orbits, the frontal bones show a temporal line, which joins the sagittal crest. This fossil differs from PIMUZ A/V4656 (Fig 7A) in lacking the supraorbital ridge. There is no lacrimal fossa. Only one lacrimal foramen is apparent, but this portion of the skull is poorly preserved, and two foramina may have been present. The frontal bones are flat in shape.

#### Nuchal region

The supraoccipital bone is high and narrow. A very strong sagittal crest joins the preeminent nuchal crests to form a knot. The nuchal crests are preeminent and backwardly projected, like in the early bovid *Eotragus* Pilgrim, 1939 [81], but are less marked than in the early crown Pecora as in *Dremotherium* Geoffroy Saint-Hilaire, 1833 [82] and *Bedenomeryx*. The paroccipital process is prominent, extending ventrally.

#### Basicranial region

The basioccipital bone is of triangular outline in palatine view, elongate, and exhibits two weakly distinguished muscular tuberosities. Like in *Eotragus*, the basioccipital bones and the basisphenoid are slightly tilted ventrally compared to the rest of the skull axis. In comparison, the stem cervid *Procervulus* Gaudry, 1878 [83], for example, has more horizontally arranged basioccipital and basisphenoid bones.

### Petrosal bones (Fig 9)

*Ventrolateral surface*. The promontorium shows two different portions: the caudal one with an unusually bulged drop-like shape and strong medio-lateral elongation, and the much flatter rostral part separated by a deep valley-like structure. The epitympanic wing is very broad and has a rather blunt apex. The caudomedial flange of the promontorium is widely expanded and continuous with the epitympanic wing. A faint but wide and curved transpromontorial sulcus is visible on the flat part of the promontorium. The fenestra cochleae (i.e., round window) is much larger, at least twice as large as the oval-shaped fenestra vestibuli (i.e., oval window). The former is slightly more caudally positioned than the fenestra vestibuli and faces laterally. A broad crista interfenestralis separates both windows. The fossa for the tensor tympani muscle is very large, taking up about half the size of the promontorium. It is round to bean-shaped and protrudes into the tegmen tympani. A large secondary facial foramen is visible next to the fenestra vestibuli and posterior to the fossa for the tensor tympani. It opens on the wide facial sulcus separated from the round window by a large, high, and thick wall of the promontorium. A rather elongated stapedial muscle fossa is also clearly marked. The ventrolateral tuberosity is large.

#### Dorsolateral surface

The tegmen tympani is inflated and has an elongated triangular shape. Its surface is rather smooth. The rostral process of the tegmen tympani is short and blunt. The hiatus Fallopii is a very distinct hole at the base of a long and wide groove emanating from the promontorial side of the petrosal bone.

#### Dorsomedial surface

No prefacial commissure fossa (sensu O’Leary [84]) is visible. The internal acoustic meatus sits in the middle of the surface and shows both foramina acusticus (superius and inferius) separated by a thin crista transversa. A basicapsular groove appears to run on the lateral side of the petrosal (or “occipital surface” sensu Mallet and Guadelli [85]) down to more or less the level of the internal acoustic meatus. The subarcuate fossa is shallow, as in all pecoran ruminants, and wide. The opening for the vestibular aqueduct lies lateral to the subarcuate fossa. It is a large slit situated posteriorly to the cochlear aqueduct lying on the ventromedial surface of the petrosal bone. The mastoid region is very large and wedge-shaped.

#### Ventromedial surface

The basicapsular groove is visible and it is more dorsal than ventral. It runs down to the cochlear aqueduct. The latter is a large and distinct hole linking four grooves: the basicapsular groove coming from the apex, two grooves coming from the ventrolateral surface (mastoid fossa) and from the dorsomedial surface, and one from the mastoid area.

### Bony labyrinth (Fig 9)

The cochlea makes 2.75 turns. The aspect ratio (total height divided by total width) is 0.55 classifying the cochlea as a rather high one (sensu Gray [86]). The cochlear spiral is tight; the turns are relatively thin and asymmetric in section. The second turn is slightly keeled. The apical turn is rather flat. The secondary bony lamina runs on the dorsal surface of the basal turn over more than one turn. The basal turn begins caudal of the fenestra cochleae and shows a slight lateral expansion on the scala tympani. The basal turn is thus slightly inflated. The cochlear aqueduct is long and thin with a circular cross section. It ends in a thick triangular pouch-like structure. Both fenestrae are well visible. The relatively small fenestra cochleae is slightly more posteriorly positioned than the fenestra vestibuli. The fenestra vestibuli is not very ellipsoid in shape, with a stapedial ratio of 1.42 (long axis divided by short axis). On the vestibule, both the utriculus and sacculus are well defined, the sacculus being round in shape while the utriculus is elongated and ellipsoid in shape. The anterior semicircular canal is the most dorsally expanded canal; it expands above the level of the common crus while the posterior semicircular canal extends only slightly above the common crus. The common crus is straight and elongated. The lateral semicircular canal is straight over its entire course even close to the posterior ampulla. It branches on the posterior semicircular canal creating a short secondary common crus, a characteristic seen in basal artiodactyls [87], but also in basal pecoran ruminants. The ampullae at the base of the semi-circular canals are well defined and globular. The vestibular aqueduct is a rather thick canal detached from the common crus. It originates at the base of the common crus in a thick pear-like insertion, and very much anterolaterally to it. It runs up the dorsal most extent of the common crus. The endolymphatic sac at the end of the vestibular aqueduct is a small thin and flat triangular structure ending at about the dorsal most extent of the anterior semicircular canal.

### Mandible (Figs 10 and 11)

The corpus is generally the best-preserved part of the mandibular remains (Figs 10 and 11). This portion is deep and massive. It regularly enlarges from p2 to m3. The masseteric fossa is shallow and flat, marked by a barely distinguishable rostral ridge (Fig 10A). The mandibular foramen is large and deep. It is located on the ramus in line with the molar row. A curved mylohyoid groove crosses a deep and well-marked pterygoid fossa. On the medial part of the mandible, between the corpus mandibularis and the mandibular angle, there is a fairly prominent bony crest for the insertion of the masseteric muscle. The space between the m3 and the ramus mandibulae space may be short. The incisura vasorum is marked and rounded. The corpus is comparatively large and massive, with a slightly concave margo ventralis. In occlusal view, the mandible narrows at the level of the diastema, just rostral of p2. The large and oval mental foramen is located at the level of the mandibular symphysis. The long diastema separating the canine and p2 forms a sharp crest dorsally (Figs 10 and 11). The lingual groove covers the caudal half of the mandible from m3. The ramus is large and oriented dorsal to subdorsal. The coronoid process is quite large and straight. The condylar process is in the axis of the ramus in the ontogenetically youngest and slightly off-centred in individuals of older individual age. There is a clear incisura mandibulae between the condylar process and the beginning of the coronoid process. The coronoid process is well rounded.

### Lower dentition (Figs 10 and 11)

#### Deciduous lower dentition

di1 is slightly rounded in its apical portion and is the smallest incisor. di2 is larger, slightly rounded in its posterior part, and straight in its anterior part. di3, the largest incisor, is procumbent, trapezoid, and strongly asymmetric with the posterior lateral section being the longest. The d2 and d3 resemble their successors in the permanent dentition (p2 and p3), d3 being longer than d2. The mesolabial conid is the highest cusp and centrally situated; and this mesolabial conid is more labial on d3. The anterior conid forms the anterior edge of the tooth. The transverse cristid is lingual, short, and posteriorly oriented on d2. It connects the mesolabial conid to a small oblique mesolingual conid on d3. The posterolabial cristid is divided into the oblique posterolingual conid, which reaches the labial part of the tooth, and the posterior and labially oriented stylid, which constitutes the posterior part of the tooth. A narrow back valley is formed between the posterolingual conid and posterior stylid. There is no cingulid. The d4 is trilobed and fully selenodont. The posterior fossa is the largest one. Neither the prehypocristid nor the posthypocristid connect the entocristids. The prehypocristid is simple and anteriorly oriented, whereas the posthypocristid is bifurcated and labially oriented. The posterior bifurcation of the posthypocristid forms a well-developed entostylid. The protoconid is well developed. Between the protoconid and the hypoconid, there is a large ectostylid forming a diagonally elongated column. There is no external postprotocristid. The internal postprotocristid is labially oriented. The preprotocristid is anteriorly oriented. The latter does not join the premetacristid, but it can be fused with the posterior cristid of the anterolabial conid. The labial cuspids are relatively aligned together. The metaconid is smaller than the entoconid, but it possesses the same orientation. The metaconid rib is well formed, and the metacristids are straight. The premetacristid ends within the anterior fossa. Between the protoconid and the anterolabial conid, the anterior ectostylid is large. The anterior cristid of the anterolabial conid joins the anterior cristid and the anterolingual conid, forming an angle on the anterior part of the tooth. The anterolingual conid is flat and anteroposteriorly oriented. There is no cingulid.

#### Permanent lower dentition

The enamel of the tooth crown is wrinkled. p1 is absent. p2-4 have nearly the same occlusal pattern, the premolars being more molarized and more elongate from p2 to p4. They are triangular in labial view due to a prominent posterolabial conid, well distinguished, with a groove on its anterolabial part. p2 lacks the anterior conid and the mesolingual conid. On p2-3, the posterior stylid is absent. The posterolabial conid becomes smaller from p2 to p4, whereas the mesolabial conid becomes larger. The mesolabial conid is the highest cusp of the premolar, central, but generally poorly distinguished. The anterior stylid and the anterior conid are well developed on p3-4. On p4, the mesolingual conid is barely distinguishable, with a well-developed backwardly-oriented transverse cristid. The transverse cristid, the posterior cristid and the posterior stylid are all in parallel. Anterolingual and posterolingual cristids are absent. The anterior valley is large and lingually open, and the posterior valley is deep. The anterior valley often possesses a lingual cingulid.

The molars are selenodont with relatively high cuspids on unworn teeth compared to other contemporaneous pecorans. The enamel is wrinkled. All the lingual cuspids are aligned forming a relatively flat lingual wall. There is a very weak metastylid. The protoconid, the hypoconid and the hypoconulid are large. The anterior and posterior fossae can be lingually closed by the preprotocristid joining the premetacristid and the posthypocristid joining the postentocristid respectively. No external postprotocristid can be observed. The prehypocristid is relatively transverse, and touches neither the entoconid nor the internal postprotocristid. The posthypocristid is relatively labio-lingually oriented. The ectostylid is well individualized, forming a small column between the protoconid and the hypoconid. Its base can be circular, ovoid, or elongated. The entoconulid is a well-developed and individualised cusp. It is as large as the metaconid and the entoconid, forming a double crescent third lobe of m3. The back fossa of m3 is bounded by a high posthypoconulidcristid delimitating a large basin. The molar crown has a well-marked anterior cingulid.

### Upper dentition (Figs 4–8)

#### Deciduous upper dentition

The upper deciduous premolars are relatively compact and molarized in missing an anterior cone associated to an anterolingual crista. The size of the protocone and the metaconule widely differs, D2 having both cusps reduced with a larger metaconule than the protocone, D3 having only the metaconule, and D4 having both cusps well developed giving the tooth a molar form. Regarding the cristae, there is a clear complexification of the crest pattern from D2 to D4. The preprotocrista extends labially and tends to reach the parastyle. On D4, the postprotocrista can be bifurcated. Between the protocone and the metaconule, the entostyle is relatively high. The premetaconulecrista is always bifurcated on D4 and sometimes on D3 as well. These crests remain, however, isolated without any fusion with other crests. A metaconule fold, antero-posteriorly oriented, crosses the posterior fossa on D3 and D4 from the posterolabial part of the metacone to the center of the postmetaconulecrista. The labial wall is relatively flat. The paracone is well developed and possesses a labial rib with an anterior groove. The metacone becomes larger on D4 and its labial side displays a weak rib. The parastyle and the metastyle are relatively globular and salient. However, only the D4 possesses a mesostyle, which is extremely reduced.

#### Permanent upper dentition

The upper canine is long and regularly curved. Its antero-posterior section is triangular at its apex. The upper premolars share a similar basic morphology, but are increasingly molarised from P2 to P4. In this context, they increase in length and width from P2 to P4, and change from an ovoid shape in P2, a half-moon shape in P3, to a more triangular shape in P4. The anterolabial cone is the highest cone, becoming more central from P2 to P4, while the lingual cone increases in size from P2 to P4, and becoming more crescentic on P4. The anterolabial crista becomes more anteriorly elongated associated to a less prominent anterolabial cone ridge. The anterior style is salient, but weak. A posterior style is absent. The posterolabial cone is well formed on P3, as large as the anterolabial cone, and less visible on the other premolars. The posterolabial crista and the posterolingual crista do not fuse, leaving a gap on the posterior part of the teeth. The central fold joins the middle part of the posterolingual crista to the center of the lingual cone framing a large basin separated from the fossa by this fold, the anterior basin being larger.

The upper molars increase in size, but decrease in complexity from M1 to M3. The pre- and postprotocrista are labially oriented. On M2-3, the preprotocrista reaches the parastyle. The premetaconulecrista never fuses with the postprotocrista. The latter is generally labially oriented with a distal end anteriorly oriented section. The premetaconulecrista is also oblique and ends between the metacone and the paracone by means of a bifurcation, which remains unconnected to any adjacent cusp and crest. The bifurcation becomes less prominent on M2 and M3. The postmetaconulecrista is labially oriented and it reaches the base of the metastyle. The metaconule fold becomes less marked from M1 to M3. The metaconule fold can be isolated, connected, or bifurcated. On the anterior part of the metaconule, the entostyle forms a rounded small column, which may be elongated. The metacone and paracone are well developed, relatively flattened labially, without prominent ribs. The labial side of the metacone-complex becomes more concave from M1 to M3. The metastyle does not form a small column. The mesostyle and the parastyle are small and sharp. The pre- and postmetacrista are slightly curved and join the mesostyle and the metastyle respectively. The post- and preparacrista are straight and aligned.

## Phylogenetical results

PAUP recovered 39 equally parsimonious trees (MPT) of 230 steps, a consistency index (CI) of 0.417 and a retention index (RI) of 0.575. The distribution of character states for the internal nodes and the list of autapomorphies are presented in the S1 Data.

**Fig 12:**
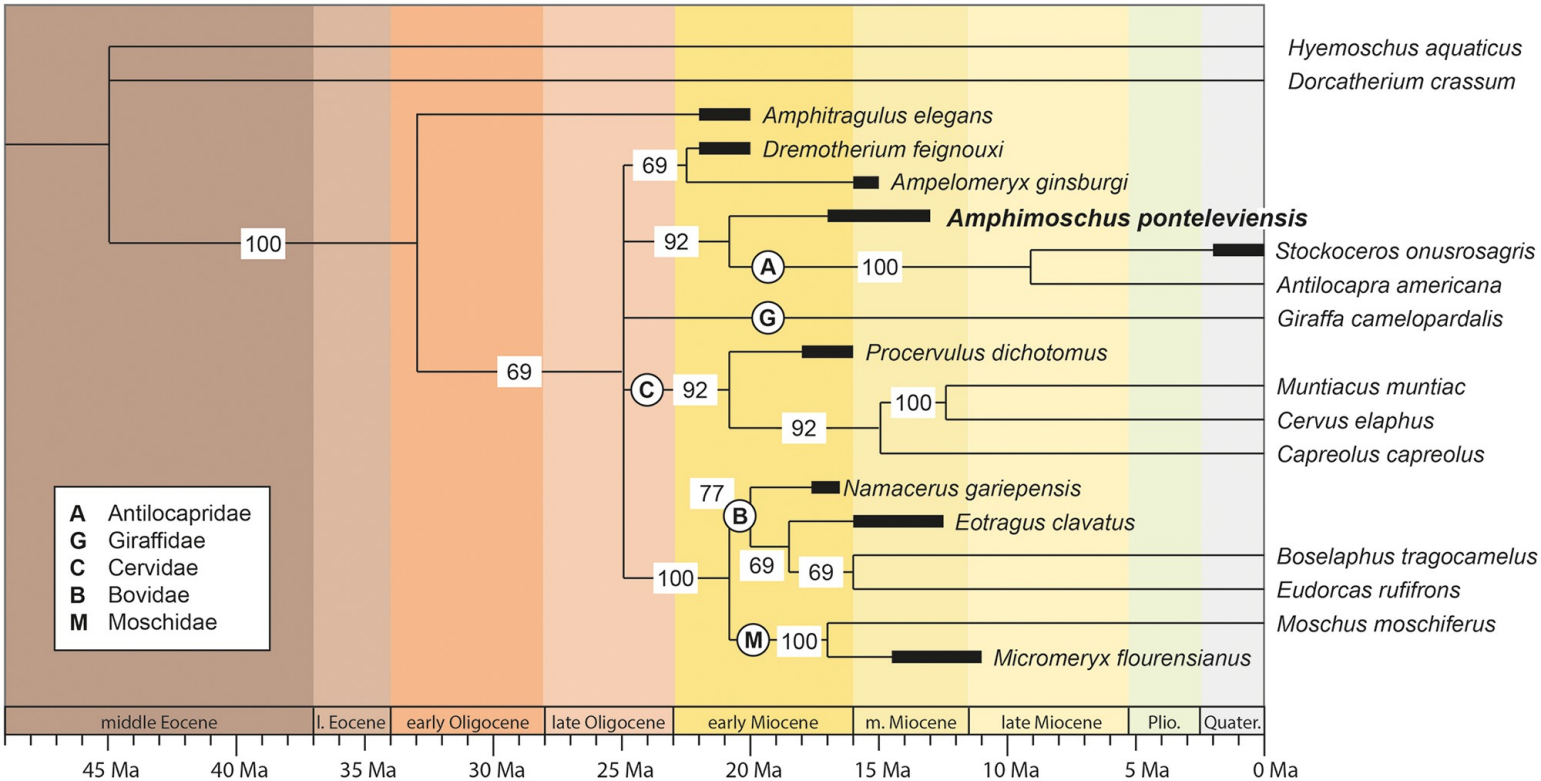
Phylogenetic tree of the majority rule consensus (50%) highlighting the systematic position hypothesis of *Amphimoschus* within the Pecora. The number on the branches indicates the rate of recovery of the clade. 39 equally parsimonious trees (MPT) with a best score of 230 steps were recovered, with a consistency index (CI) of 0.417 and a retention index (RI) of 0.575.

The strict consensus is poorly resolved with a basal polytomy from which only the clade bovids+moschids is preserved. Thus, we here discuss the majority rule consensus (50%) (Fig 12): the values on the branches corresponding to the frequencies for all observed bipartitions. In the majority consensus, *Amphitragulus* is basally placed as a sister taxon of the rest of the in-group as in Sánchez et al. [44] and Mennecart et al. [51]. Then, we observe a general polytomy separating a clade formed by *Dremotherium* + *Ampelomeryx*, Antilocapridae (extant and fossil) + *Amphimoschus*, *Giraffa*, Cervidae (extant and fossil), and Bovidae (extant and fossil) + Moschidae (extant and fossil). Here, *Ampelomeryx* is not grouped with *Giraffa* within the Giraffomorpha, as opposed to Sánchez et al. [44] and Mennecart et al. [51], and *Dremotherium* is not closely related to the “Cervidae” [44] nor to the Bovidae + Moschidae [51]. The clades (*Moschus* + *Micromeryx*) and (bovids + moschids) are well supported. *Amphimoschus* appears as a sister group of the well supported clade encompassing *Antilocapra* and *Stockoceros*. The position of *Amphimoschus* close to antilocaprids is supported by 12 synapomorphies (S1 Data), even though four of them are not coded for *Amphimoschus* (characters 19, 53, 54, and 56). *Amphimoschus* possesses three autapomorphies: the anterior position of the maxilla-palatine-suture line (21), the position of the foramen ovale with respect to the infratemporal fossa (39), and the bicuspidate third lobe of the m3 (44, constrained as irreversible in the analysis). The clade Antilocapridae is also supported by 12 synapomorphies (characters 17, 18, 22, 23, 25, 28, 34, 40, 46, 47, 50, and 51; see S1 Data).

## Discussion

### How many species of Amphimoschus?

Two species of *Amphimoschus* are frequently cited in the European Orleanian and earliest Astaracian deposits: *Amphimoschus ponteleviensis* Bourgeois, 1873 [4] from Thenay Sables et Marnes du Blésois (MN5a) (misattributed to Thenay Sables de l’Orléanais Formation MN4 [4] and Pontlevoy Falun Formation MN5b [9]) and *Amphimoschus artenensis* Mayet, 1908 [9] from Artenay (MN4a). The stratigraphic range of *Amphimoschus artenensis* covers the early Orleanian whereas that of *A*. *ponteleviensis* includes the late Orleanian to earliest Astaracian [16,32,35]. The distinction between these two species is based on the height of the crowns, the shape of the ectostylid base, the more bulged metaconid and the overall tooth-size [9,16,64]. It is interesting to note that Mayet [9] published significantly larger measurements of the type specimens from Thenay than what we observed, with a discrepancy in size of 1 to 3 mm (for example MNHN.FP.876 m1: 12.2 vs 15; MNHN.FP.1070 p3: 10.1 vs 13, the first measurement being our data and the second from Mayet [9] respectively).

Based on wear features of the tooth crowns, the large species, *A*. *ponteleviensis*, was classified as a browser, while the smaller (*A*. *artenensis*) was considered to be a mixed feeder [35]. Stehlin [18] had previously explained, that the size difference between the two species are not that clear, as there are huge size overlaps when considering larger samples of the two “mutations”, as he puts it. However, in absence of further material, no conclusion was drawn.

In this study, the teeth of 250 specimens have been measured (S2 Data) and 780 teeth have been observed at the locality level (S3 Data). Tooth morphology varies little within an assemblage (i.e. a horizon of a fossil locality). For example, the morphology of the ectostylid can be highly variable (column with a circular, triangular, or even extended shape) in a same locality. The postentocristid and the posthypocristid may or may not be fused depending on the age of the individual and the wear state of the teeth. Likewise, the fusion of the premetacristid and preprotocristid may vary in an assemblage. The cranial characters remain difficult to interpret due to different ontogenetic stages and no clear sexual distinction.

The lower m3 is the easiest recognisable tooth (because of the uncommon feature of a double crescent in the third lobe). This is the most frequently identified tooth, with 87 specimens observed by the late Léonard Ginsburg when he started his unfinished study of *Amphimoschus*. Regarding size variation, important overlaps exist between fossil materials from different biochronological zones from MN3 to MN5. In this study (S2 Data), we compared 65 third molars from different localities and ages. The results of the ANOVA show that the *Amphimoschus* chronological assemblages cannot be distinguished based on the length of the m3 from the three biozones: MN3, MN4, and MN5 (*p =* 0.5586; main variance differences being observed comparing the data from MN5 to the other ages). Tooth crown height and metaconid shape are also similar across the whole dataset, we could not observe the differences that Mayet [9] reported. Accordingly, size differences and tooth morphology cannot provide an empirical basis to justify the validity of both species. Hence, we conclude that there was only one species of *Amphimoschus*, which roamed Europe during the late early to the middle Miocene. The nomenclatural priority goes to *Amphimoschus ponteleviensis* Bourgeois, 1873 [4].

Looking at the size and shape of the teeth, we agree with Stehlin [18] in attributing the specimens ascribed to *Cervus lunatus* by von Meyer [19] from Käpfnach (MN5) to *A*. *ponteleviensis*. Mayet [9] mentioned the presence of an *Amphimoschus* most likely similar to the smaller form from Artenay in Neuville aux Bois (MN3). Unfortunately, the specimen (MSNO 224) was lost during the early 20^th^ century [9]. He also mentioned a specimen “b” not included in our analysis since the published sizes are much larger than observed in *Amphimoschus ponteleviensis*. Raia *et al*. [88] referred to an “*Amphimoschus elegans*” based on their geometric morphometrics analysis. This undescribed species might actually be the result of confusion between the genera *Amphimoschus* and *Amphitragulus*. The mandibles of these genera are different in shape [6]. The mandible described by Raia *et al*. [88] looks very similar to those of *Amphitragulus elegans* of Mennecart *et al*. [6].

Based on this information, only the species *Amphimoschus ponteleviensis* Bourgeois, 1873 [4] should be considered as valid in the European fossil record.

### Ontogeny of Amphimoschus

The studied crania belong to different ontogenetic stages. The youngest one (SMNS 45592, Fig 6) only possesses a d4, a D4 and an erupting M1. No wear can be observed on the occlusal surface of teeth. By comparing to age classes from living ruminants, the specimen would not be older than three months [89]. In another specimen (SMNS 40693, Fig 4), all deciduous premolars and molars are preserved and the m3 is erupting, suggesting they are from an animal in its second year. The sagittal crest of the latter is not as developed as in specimens belonging to older individuals (e.g., MNHN.F.Ar3266, Fig 8). In specimen PIMUZ A/V4656 (Fig 7A), all the molars are heavily worn. In addition, this cranium possesses some unique features. Both P4s are missing, which could be due to a palaeopathology. The teeth may have been retained in a bud stage, or they were already lost due to the advanced age of the individual. However, the bilateral absence of these two teeth and the reduction of the gap between P3 and M1 may indicate the original absence of these structures. Moreover, this specimen has very strong supraorbital ridges, which are absent in the other specimens. In extant ruminants, such a feature is only observed in *Muntiacus* Rafinesque, 1815 [90] and may be associated with cranial appendages. However, so far no cranial appendages have been found associated with *Amphimoschus*.

### Phylogenetic position of Amphimoschus

The absence of cranial appendages in the described *Amphimoschus* crania makes its phylogenetic affinities uncertain. All authors are in agreement about pecoran affinities based on the fully fused metapodial bones (observed in Bourgeois 1873 [4]), massive and complex p4, relatively high crowned molars in comparison to the contemporaneous European ruminants, fully selenodont teeth, and the shallow subarcuate fossa on the petrosal. However, its phylogenetic position within pecoran ruminants remains highly debated. Bourgeois (1873) [4] described *Amphimoschus* as closely related to the hornless *Moschus* (Moschidae). However, except for *Hydropotes inermis* (the only living ruminant where the absence of appendages is known as secondarily lost), the absence of cranial appendages is a plesiomorphic character.

*Amphimoschus* is recovered in our phylogenetic analysis as the sister taxon of the Antilocapridae (the pronghorns). This family is known by a relatively rich Neogene fossil record in North America where the family is known today by only a single extant representative (*Antilocapra americana*) [91]. *Paracosoryx* and *Merycodus*, the earliest known antilocaprids, are dated to ca. 18.8 My [91] which is about the age of *Amphimoschus* occurrences in Europe (see Table 1). Since no older Pecora have been found in Northern America, it is admitted that Antilocapridae most likely originated in the Old World and subsequently migrated to North America [91]. Similarly to extant and fossil Antilocapridae, *Amphimoschus* possesses a short vestibular aqueduct (15), a lateral semicircular canal partly fused with the posterior ampulla (16), absence of contact between the postglenoid process and the external acoustic meatus (27), a small postglenoid foramen (28), the parieto-squamosal suture located in the middle of the upper and lower borders of the temporal fossa (36), a highly developed postentocristid that fuses with the posthypocristid, distally closing the lower molars (43), and a bicuspidate third lobe of the m3 (45). However, we refrain in our Systematic Paleontology section from classifying *Amphimoschus* with Antilocapridae. A short vestibular aqueduct may be convergent in different lineages [55], while the relatively low position of the lateral semicircular canal is found in Pecora other than crown Bovidae and crown Moschidae [43,56,92]. It has recently been shown that the morphology of the bony labyrinth bears a strong phylogenetical signal in artiodactyls [55,93–95]. This bony structure is species-specific, and varies little [96,97]. Very little is currently known on the bony labyrinth morphology in Miocene ruminants [42,43,55,56,92]. *Amphimoschus* shows a mosaic of apomorphic and plesiomorphic traits of its bony labyrinth with the plesiomorphic condition of a decentred vestibular aqueduct, but the derived features of the cochlea showing a high number of turns (0.75 turns more than stem early Miocene cervids, see [55,56]). The cochlea, which is detached from the vestibule (not seen in stem cervids, see [55,56]), has turns of roughly the same thickness (again see [55,56] for a contrasting condition in stem cervid). Moreover, all the cranial characters uniting *Amphimoschus* with the Antilocapridae can be observed in other lineages (27/1 in Giraffoidea and Bovidae, 28/0 Giraffoidea and *Procervulus*, 36/0 in Cervidae and Bovidae). Leinders [20], Janis and Scott [5], McKenna and Bell [22], and Mazza and Rustioni [21] saw a close relationship of this genus with the enigmatic *Hoplitomeryx* Leinders, 1983 [20] (late Miocene-Pliocene, Gargano, Italy), based on the shape of the tympanic bullae, the bicuspidate third lobe of m3, and the morphology of the p4. Van de Geer *et al*. (page 78 [98]) state, there is not “[…] any evidence to consider the hornless extinct moschids *Micromeryx* and *Amphimoschus* as sister-taxa of *Hoplitomeryx*”. They argue that “*Hoplitomeryx* differs from this genus [*Amphimoschus*] owing to the presence of cranial appendages, loss of lower p2, a non-bifurcated protocone, weakly developed entostyle and ectostylid“. Likewise, the morphology of the third lobe of the m3, (bicuspidate and with well-developed post-entoconulidcristid) has appeared convergently in *Amphimoschus*, *Hoplitomeryx* spp., *Lagomeryx pumilio*, and moschids. Having more hypsodont teeth to cope with more abrasive food items may induce a reduction or loss of the external postprotocristid (*Palaeomeryx* fold) [5], the absence of a p1, and a robust corpus mandibularis [6,99] which are all also observed convergently in *Amphimoschus*, Antilocapridae, and Bovidae. Consequently, the position of *Amphimoschus* suggested by our phylogenetic analysis should be taken with caution. Moreover, despite the backbone phylogenetic tool applied on extant species and the Miocene Cervidae *Procervulus*, the relative systematic position of the different Crown pecoran families appears poorly resolved. If the molecular phylogenies of pecoran ruminants are congruent (e.g. [47–49]), those based on the morpho-anatomical characters remain conflicting with the former. This is accentuated when fossils are included in the phylogenetic analysis, suggesting that convergent evolution is important among pecorans: Moschidae as a sister taxa of Cervidae in a supertree [50]; Antilocapridae closely related to Moschidae and Bovidae in a Bayesian analysis [44]; Cervidae as the basalmost pecoran in a heuristic analysis [51]. Hence, the phylogenetic position of the early Miocene and hornless Pecora remains problematic. Our phylogenetic hypothesis will have to be tested with a larger dataset, including additional taxa and new morphological features, especially for numerous extinct taxa that remain poorly documented.

## Supporting information

S1 Data Description of the character states and of the character matrix of the phylogenetical analysis with associated results. (ZIP)

S2 Data Measurements of European *Amphimoschus ponteleviensis*. (XLSX)

S3 Data Measurements of European *Amphimoschus ponteleviensis* at the locality level. Most of the data has been provided by our late colleague, L. Ginsburg on unreferred material.
(XLSX)
