# Supplementary material for: Reassessment of the enigmatic ruminant Miocene genus Amphimoschus Bourgeois, 1873 (Mammalia, Artiodactyla, Pecora)
Source: PLoS One. 2021 Jan 29;16(1):e0244661. doi: 10.1371/journal.pone.0244661 (PMC7846017; doi:10.1371/journal.pone.0244661)
Supplement: S1 Data — (ZIP) [file pone.0244661.s001.zip › R1_Supplementary data 1/Amphimoschus_Phylogeny_character_list.docx]

**Petrosal Bone**

1. Shape of the fossa for the tensor tympani muscle: round to square (0); elongated and bean-shaped (1) (from [[1](#_ENREF_1)]). Aiglstorfer-1.
2. Extent of the fossa for the tensor tympani muscle: not excavated into the tegmen tympani (0); excavated into the tegmen tympani (1) (from [[2](#_ENREF_2)]). Aiglstorfer-2.
3. Transpromontorial sulcus: present (0); absent (1) (from [[2](#_ENREF_2)]). Aiglstorfer-3.
4. Medial protrusion of the pars cochlearis: absent (0); present (1) (from [[2](#_ENREF_2)]). Aiglstorfer-4.
5. Knob anterior to the subarcuate fossa: absent (0); present (1) (from [[1](#_ENREF_1)]). Aiglstorfer-5.
6. Subarcuate fossa: shallow (0); deep (1) (from [[2](#_ENREF_2)]). Aiglstorfer-6.
7. Shape of mastoid region: wedge (0); knob (1) (from [[2](#_ENREF_2)]). Aiglstorfer-7.
8. Basicapsular groove: absent (0); dorsal (1); on the edge of the epitympanic wing (2) (modified from [[2](#_ENREF_2)]). Aiglstorfer-8.
9. Anterior process of the tegmen tympani: not protruding (0); protruding (1) (this study). Aiglstorfer-9.
10. Anterior process of the tegmen tympani: broad and ovoid (0); pointed pilar (1); broad and triangular (2) (this study). Aiglstorfer-10.

**Bony Labyrinth**

1. Number of cochlear turns: equal or less than two (0); between two and three (0); equal or more than three (1) (modified from [[3](#_ENREF_3)]). Aiglstorfer-11.
2. Insertion of the lateral semicircular canal in the vestibule towards the posterior ampulla: low in posterior ampula (0); high dorsally between posterior ampula and commun crus (1); high in posterior ampula (2); anterior to posterior ampula in vestibule (3) (modified from [[3](#_ENREF_3)]). Aiglstorfer-12.
3. Extension of the lateral semicircular canal with respect to the plane of the posterior semicircular canal in dorsal or lateral view: no extension beyond the plane (0); extension beyond the plane (1) (modified from [[3](#_ENREF_3),[4](#_ENREF_4)]). Aiglstorfer-13.
4. Course of the vestibular aqueduct with respect to the common crus: parallel (0); diverging (1) (from [[1](#_ENREF_1)]). Aiglstorfer-15*.*
5. Length of the vestibular aqueduct: less than the common crus (0); same as common crus (1); longer than common crus (2) (from [[1](#_ENREF_1)]). Aiglstorfer-16.
6. Fusion of the lateral semicircular canal with posterior ampulla: absent (0); partial to complete fusion (1) (from [[5](#_ENREF_5)]). Aiglstorfer-18.
7. Section of the cochlear aqueduct: incipious (0; )flat (1); ovoid to circular (2) (from [[5](#_ENREF_5)]). Aiglstorfer-20.
8. Shape of the endolymphatic sac: straight and funnel-like (0); triangular in shape (1); pouch-like (2) (from [[1](#_ENREF_1)]). Aiglstorfer-21.

**Cranial**

1. Fontanella nasolacrimalis (ethmoidal vacuity): absent (0); small, less than 2/3 of the facies facialis of the lacrimal (1); large, 2/3 of facies facialis of lacrimal or more (2) (modified from [[6](#_ENREF_6)]; character-list 1). Aiglstorfer-34)
2. Fossa lacrimalis: present (0); absent (1) (modified from [[6](#_ENREF_6)]; character-list 1). Aiglstorfer-35)
3. Maxilla-palatine-sutureline on bony palate: reaching as anterior as or more anterior than P4 (0); reaching not more anterior than M1 (1). Aiglstorfer-37)
4. Condition of the tympanohyal vagina: without lateral enclosing (0); reduced enclosing, affecting only the proximal third part of the tympanohyal (1 ); complete enclosing, the tympanohyal vagina is not visible in lateral view (2). Sanchez-1.
5. Position of the tympanohyal vagina in the tympanic bulla: Caudal (0); central or sub-central (1). Sanchez-3.
6. Contact between the tympanic bulla and the basioccipital: no contact (0); contact (1). Sanchez-5.
7. Lacrimal orifices: one orifice (0); two orifices, being clearly one above the other even if the upper one is more or less inside the lacrimal (1); two orifices, almost aligned (2). Sanchez-6.
8. Retroarticular process: nearly absent (0); present with no expansions (1); anteroposteriorly wide with well-developed posterior expansions (2); absent, with only a very robust lateral expansion (3); developed with very large and triangular lateral expansion (4). Sanchez-9.
9. Contact between the postglenoid process and the external acoustic meatus: no contact (0); contact (1). Sanchez-10.
10. Morphology of the postglenoid foramen: small (0); large and laterally open (1); large and laterally closed due to the expansion of the tympanic bulla (2); small and laterally open (3). Sanchez-11.
11. Antlers: absent (0); present at least in males (1). This study
12. Antlers burr: absent (0); present (1). Heckeberg 6 (Antlerogenesis paper)
13. External antler to pedicle transition: not obvious (0); obvious (1). Heckeberg 9 (Antlerogenesis paper)
14. Origination of the Antlers pedicles in lateral view: directly above orbita (0); well behind orbita (1). Heckeberg 17 (Antlerogenesis paper)
15. Ossicones: absent (0); present at least in males (1). This study
16. Pronghorns: absent (0); present at least in males (1). This study
17. Horns: absent (0); present at least in males (1). This study
18. Location of the parieto-squamosal suture: located in the middle of the upper and lower borders of the temporal fossa (0); located near the inferior border of the temporal fossa (1). Sanchez-19.
19. Condition of the lateral margin of the infratemporal fossa: poorly developed (0); well-marked (1). Sanchez-20.
20. Depth of the infratemporal fossa: shallow (0); deep (1). Sanchez-21.
21. Separation between the foramen ovale and the infratemporal fossa: separated by a low and sharp small crest (0); separated by a well-developed crest (1); not directly adjacent (2). Sanchez-22.

**Dental**

1. Condition of the upper canines of males: canines absent (0); presence of robust and wide hypertrophied canines that develops vertically with a more or less pronounced distal curvature (1); presence of long, slender and narrow saber-like canines showing a proximally concave and distally convex lateral surface (2); small canine present (*cervus*…) (3). Sanchez-39.
2. Morphology of the P4: triangular in shape (0), semicircular in shape (1) (modified from [[6](#_ENREF_6)]; character-list 1). Aiglstorfer-42)
3. Labial elements in upper molars: pyramidal (0), more pillar-shaped (1) (this study). Aiglstorfer-43)
4. Development of the postentocristid (connection hypoconid/entoconid): not developed, or poorly developed, postentocristid (0); developed entocristid, almost contacting the posthypocristid (1); highly developed postentocristid that fuses with the posthypocrisitd, distally closing the lower molars (2); fusion related with hypsodonty /hypselodonty (3). Sanchez-40.
5. Development of the postprotocrista: short postprotocrista (0); expanded postprotocrista (1). Sanchez-41.
6. Morphology of the third lobe of the m3: monocuspidate (0); bicuspidate (1). Sanchez-43.
7. Morphology of p4 anterior valley: anterior valley closed: strongly developed anterolingual cristid that encloses totally or almost totally the anterior valley and the mesolingual conid joins the transverse cristid through the posterolingual cristid (0), anterior valley open (1) (modified from [[6](#_ENREF_6)]; character-list 1). Aiglstorfer-45)
8. Morphology of the p4: without lingual elements (0); the mesolingual conid joins the cristid obliqua through the posterolingual cristid (1); as in 1 and with very developed anterolingual cristid that encloses totally or almost totally the anterior valley (2); the mesolingual conid joins directly the cristid obliqua (3); absence of cristid obliqua, and the anterolingual cristid joins the anterior wing, whereas the posterolingual cristid is poorly developed and does not reach the posterior wing (4); shortened p4 with all cuspids united into a continuous occlusal surface (5). Sanchez-35.
9. External postmetacristid: present (0), absent (1) (this study). Aiglstorfer-46)
10. External postprotocristid: present (0), absent (1) (modified from [[6](#_ENREF_6)]; character-list 1). Aiglstorfer-47)
11. Development of the anterior cingulid in the lower molars: not developed (0); weak to moderately developed (1); stronger developed, clearly invading lingual side (2); strong, fused with mesostylid (3) (modified from [[6](#_ENREF_6)]; character-list 1 and 2). Aiglstorfer-48)
12. Development of the metastylid: well developed (0); weak (1); absent (2) (from [[6](#_ENREF_6)]; character-list 1 and 2). Aiglstorfer-49)

**Postcranial**

1. Vertical development of the humeral capitulum: little developed (0); developed (1); very developed (2). Sanchez-49.
2. Morphology of the capitular facet in the radius: short and wide, without dorsal development (0); elongated and wide (1); short and wide, without palmar development (2); similar to state 2 but with a conspicuous triangular dorsal process (3). Sanchez-50.
3. Morphology of the facet for the semilunate in the radius: absence of lateral notch (0); presence of lateral notch (1). Sanchez-51.
4. Condition of the distal articular facets of the semilunate: subequal (0); the facet for the unciform is larger (1); the facet for the magnotrapezoid is larger (2). Sanchez-52.
5. Condition of the canal for the digital common artery in the metatarsal III-IV: ‘tragulid-type’, superficially placed but not in an equally fused metatarsals as in the Pecora, with poorly developed groove (0); ‘moschid-type’, superficially located, but not as superficial as in ‘3’ (1); ‘cervid-type’, very deeply located, in the mid-shaft axis of the metatarsal (2); ‘bovid-type’, extremely superficially located, almost without groove (3). Sanchez-53.
6. Location of the plantar column of the metatarsal III-IV: central (0); lateral (1). Sanchez-54.
7. Condition of the plantar facet for the navicular-cuboid in the metatarsal III-IV: sloped (0); horizontal (1). Sanchez-55.
8. Metatarsal plantar tuberosity: absent (0); well developed (1); small (2). Sanchez-56.
9. Condition of the metatarsal sulcus: distally open (0); distally closed (1). Sanchez-58.
10. Development of the astragalar distal trochlea: developed (distal half of the astragalus roughly equal to the proximal half) (0); short (distal half of the astragalus much shorter than the proximal half) (1). Sanchez-60.
11. Development of the proximo-plantomedial process of the navicular-cuboid: little developed (0); developed (1). Sanchez-61.
12. Morphology of the proximo-plantomedial process of the navicular-cuboid: without any crest (0); presence of a round-shaped crest that develops in the distal part of the plantomedial process (1); presence of a well-developed crest that does not reach the proximal region of the plantomedial process (2); presence of a highly developed crest that extends along the entire plantomedial process (3). Sanchez-62.
13. Morphology of the astragalus: non-parallel sides (0); parallel sides (1). Sanchez-65.
14. Development of the metapodial keels: not developed into the extensor region of the articular facet (0); developed into the extensor region of the articular facet (1). Sanchez-66.
